# A Bioenergetic Basis for Membrane Divergence in Archaea and Bacteria

**DOI:** 10.1371/journal.pbio.1001926

**Published:** 2014-08-12

**Authors:** Víctor Sojo, Andrew Pomiankowski, Nick Lane

**Affiliations:** 1 Department of Genetics, Evolution and Environment, University College London, London, United Kingdom; 2 CoMPLEX, University College London, London, United Kingdom; Massey University, New Zealand

## Abstract

Membrane bioenergetics are universal, yet the phospholipid membranes of archaea and bacteria—the deepest branches in the tree of life—are fundamentally different. This deep divergence in membrane chemistry is reflected in other stark differences between the two domains, including ion pumping and DNA replication. We resolve this paradox by considering the energy requirements of the last universal common ancestor (LUCA). We develop a mathematical model based on the premise that LUCA depended on natural proton gradients. Our analysis shows that such gradients can power carbon and energy metabolism, but only in leaky cells with a proton permeability equivalent to fatty acid vesicles. Membranes with lower permeability (equivalent to modern phospholipids) collapse free-energy availability, precluding exploitation of natural gradients. Pumping protons across leaky membranes offers no advantage, even when permeability is decreased 1,000-fold. We hypothesize that a sodium-proton antiporter (SPAP) provided the first step towards modern membranes. SPAP increases the free energy available from natural proton gradients by ∼60%, enabling survival in 50-fold lower gradients, thereby facilitating ecological spread and divergence. Critically, SPAP also provides a steadily amplifying advantage to proton pumping as membrane permeability falls, for the first time favoring the evolution of ion-tight phospholipid membranes. The phospholipids of archaea and bacteria incorporate different stereoisomers of glycerol phosphate. We conclude that the enzymes involved took these alternatives by chance in independent populations that had already evolved distinct ion pumps. Our model offers a quantitatively robust explanation for why membrane bioenergetics are universal, yet ion pumps and phospholipid membranes arose later and independently in separate populations. Our findings elucidate the paradox that archaea and bacteria share DNA transcription, ribosomal translation, and ATP synthase, yet differ in equally fundamental traits that depend on the membrane, including DNA replication.

## Abstract 2

The deepest split in the tree of life is between archaea and bacteria. We show this split can be explained by the late evolution of impermeable membranes, for energetic reasons.

## Author Summary

The archaea and bacteria are the deepest branches of the tree of life. The two groups are similar in morphology and share some fundamental biochemistry, including the genetic code, but the differences between them are stark, and rank among the great unsolved problems in biology. The composition of cell membranes and walls is utterly different in the two groups, while the mechanism of DNA replication seems unrelated. We address a specific paradox, giving new insight into this deep evolutionary split: membrane bioenergetics are universal, yet the membranes themselves are not. We resolve this paradox by considering the energetics of a hypothetical last universal common ancestor (LUCA) in geochemically sustained proton gradients. Using a quantitative model, we show that LUCA could have used proton gradients to drive carbon and energy metabolism, but only if the membranes were leaky. This requirement precluded ion pumping and the early evolution of phospholipid membranes. We constrain a pathway leading from LUCA to the deep divergence of archaea and bacteria on the basis of incremental increases in free-energy availability. We support our inferences with comparative biochemistry and phylogenetics, and show why the late evolution of modern membranes forced divergence in other traits such as DNA replication.

## Introduction

Reconstructing the traits of the last universal common ancestor (LUCA) requires constraining the relationships between the three domains of life, the archaea, bacteria, and eukaryotes. Recent phylogenetic studies show that eukaryotes are secondarily derived: they are genomic chimeras, arising from an endosymbiosis between a bacterium and an archaeal host cell [1]–[5]. The divergence between the two primary domains, the archaea and the bacteria, is now seen as the deepest branch in the tree of life [1],[6]–[8]. The properties of LUCA are most parsimoniously those shared by bacteria and archaea. This leads straight to a serious paradox. Archaea and bacteria share core biochemistry, including the genetic code, transcription machinery, and ribosomal translation [9], but differ for unknown reasons in fundamental traits including cell membrane [10] and cell wall [11], glycolysis [12], ion pumping [13], and even DNA replication [14].

**Figure 1:**
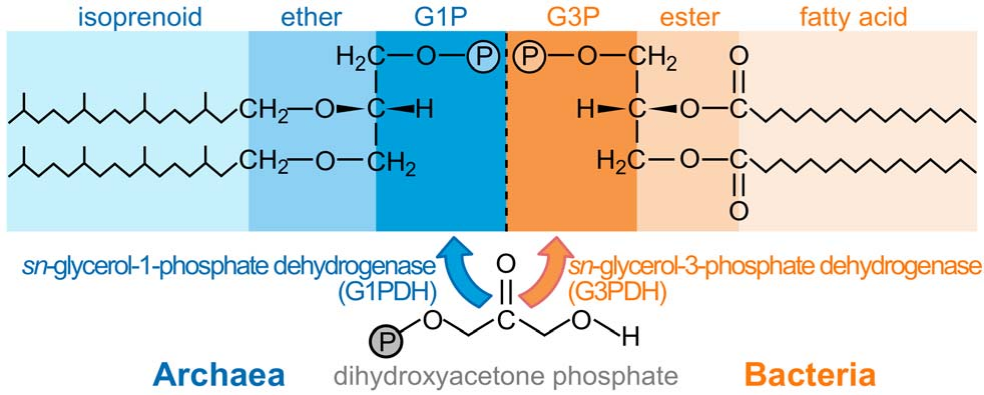
Membrane lipids of archaea and bacteria. Archaeal lipids (left) are typically composed of isoprenoid chains linked by ether bonds to an *sn*-glycerol-1-phosphate (G1P) backbone. The chirality of the two glycerol backbones is fully conserved within each clade not only in structure but in their unrelated synthetic enzymes. Although ether linkages have been observed in bacterial membranes [15] and isoprenoids are common to all three domains, bacterial lipids (right) are typically composed of fatty acids in ester linkage to an *sn*-glycerol-3-phosphate (G3P) skeleton. Despite widespread horizontal gene transfer, no bacterium has been observed with the archaeal enantiomer, or vice versa [10].

The differences in membrane lipids may be the key to this major unsolved problem in biology. Phospholipid side chains are typically isoprenoids in archaea and fatty acids in bacteria [15]. While this could reflect adaptive evolution [16], archaea and bacteria also differ in the stereochemistry of the glycerol-phosphate headgroup [10]. Archaeal lipids have an *sn*-glycerol-1-phosphate (G1P) headgroup, while bacteria use the mirror structure *sn*-glycerol-3-phosphate (G3P) (Figure 1). There is no persuasive selective explanation for these opposite stereochemistries [10],[13],[17]. The enzymes involved, glycerol-1-phosphate-dehydrogenase (G1PDH) in archaea and glycerol-3-phosphate-dehydrogenase (G3PDH) in bacteria, bear no phylogenetic resemblance, suggesting they arose independently [10]. If so, then LUCA did not possess a modern membrane—a seemingly improbable conclusion, given the central importance of membranes to cells [10],[17],[18].

Set against this paradoxical difference in membrane composition is the universality of membrane bioenergetics [19]. Essentially all cells power ATP synthesis through chemiosmotic coupling, in which the ATP synthase (ATPase) is powered by electrochemical differences in H^+^ or Na^+^ concentration across membranes [20]. The ATPase is universally conserved [21] and shares the same deep phylogenetic split as the ribosome, implying that both were present in LUCA [22]–[24]. The deepest branches in the tree of life are entirely populated by autotrophs [1],[6],[7],[12],[25], which also depend on chemiosmotic coupling to drive carbon metabolism via proteins such as the energy-converting hydrogenase (Ech) and ferredoxin [26]. But there are serious objections to the idea that LUCA was chemiosmotic. Pumping protons across membranes requires sophisticated proteins, which are only useful in membranes impermeable to protons [27]. Unlike the ATPase, no ion pumps are universally conserved [13]. The pathways for heme and quinone synthesis (the major cofactors of respiratory proteins) also differ in archaea and bacteria, although their distribution is complicated by lateral gene transfer, as is reconstruction of the phylogenetic origins of respiratory ion pumps [13]. But it seems likely that both lipid membranes and active pumping are evolutionarily distinct in archaea and bacteria [9],[11]. It is hard to reconcile these fundamental differences with the universality of the ATPase. On the face of it, LUCA was chemiosmotic, yet did not have a modern phospholipid membrane or active ion pumps.

A possible resolution is that LUCA exploited natural (geochemically sustained) proton gradients [18],[28],[29]. However, the hypothesis that natural proton gradients could drive carbon and energy metabolism in LUCA, in the absence of active ion pumps, faces a serious drawback. Because fluids are electrically balanced, the transfer of H^+^ ions down a concentration gradient, from an acid solution into a cell, transfers positive charge into the cell, generating a membrane potential that opposes further influx. The system swiftly reaches electrochemical (Donnan) equilibrium, in which electrical charges and concentration differences are offset [30]. Equilibrium is death: natural proton gradients could only drive carbon and energy metabolism in LUCA if such equilibrium is avoided—in effect, if protons accumulating inside a cell can leave again. Membrane permeability could be critical to maintaining disequilibrium in any system with continuous flow, as leaky membranes impose less of a barrier to the continued flux of H^+^, OH^−^, and other ions [19].

**Figure 2:**
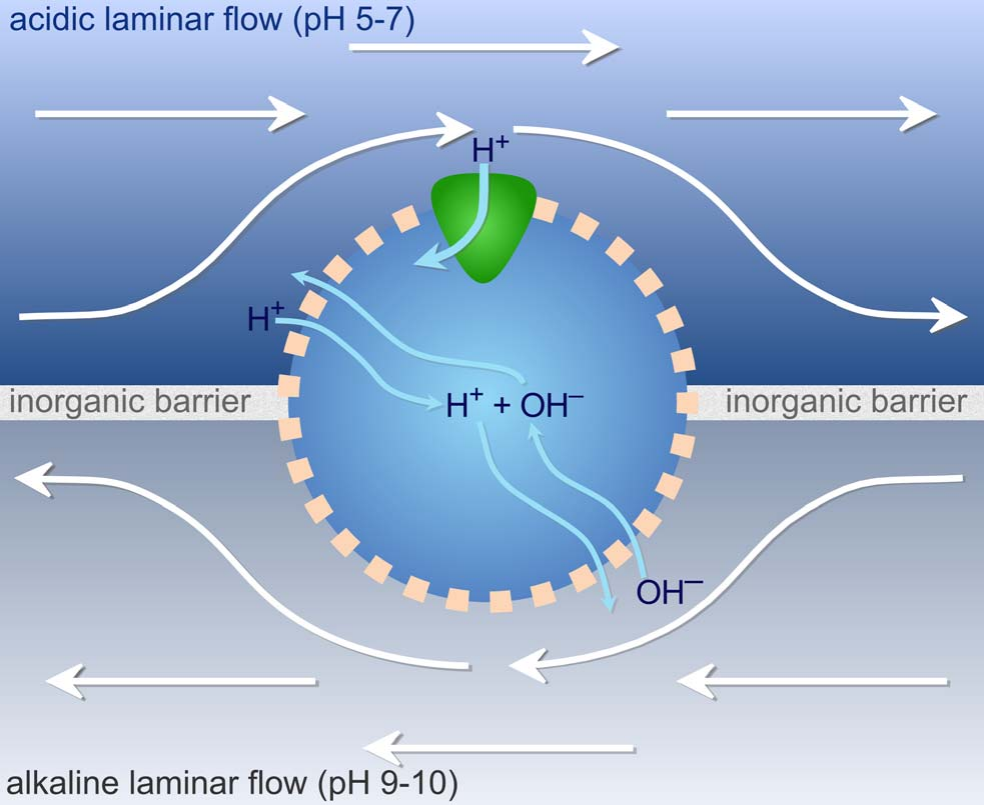
The model. A cell with a semi-permeable membrane sits at the interface between an alkaline and an acidic fluid. The fluids are continuously replenished and otherwise separated by an inorganic barrier. Hydroxide ions (OH^−^) can flow into the cell from the alkaline side by simple diffusion across the membrane, with protons (H^+^) entering in a similar manner from the acidic side. Other ions (Na^+^, K^+^, Cl^−^, not shown) diffuse similarly, as a function of their permeability, charge, and respective internal and external concentrations on each side. Inside the protocell, H^+^ and OH^−^ can neutralize into water, or leave towards either side. Internal pH thus depends on the water equilibrium and relative influxes of each ion. A protein capable of exploiting the natural proton gradient sits on the acidic side, allowing energy assimilation via ATP production, or carbon assimilation via CO_2_ fixation.

The feasibility of this hypothesis depends on the dynamics of ion fluxes that are unknown. We have therefore built a model to estimate quantitative differences in free energy (−ΔG) across lipid membranes exposed to natural proton gradients. We consider a cell exposed simultaneously to alkaline fluids and relatively acidic water (Figure 2). Our model is independent of any particular setting, but requires continuous laminar flow with limited mixing (as found in microporous alkaline hydrothermal vents [18],[19],[24],[31]–[33] and potentially other environments), allowing sharp gradients of several pH units to be maintained across short distances of 1–2 µm. In general, we assume that the external pH does not change on either side of the cell, as external fluids are replenished by continuous flow from large reservoirs (e.g., hydrothermal fluids or the ocean), but we do also consider mixing.

Protons enter the cell through membrane proteins, and directly through the lipid phase of the membrane. The overall rate of proton influx depends on the difference in proton concentration and electrical charge (upon proton entry) between the outside and inside of the cell, the kinetics of the membrane protein (e.g., ATPase), the number of membrane proteins (given as a proportion of the surface area), the proton permeability of the lipid phase of the membrane, and the rate of loss of protons from inside the cell (see Materials and Methods). For simplicity, we assume that gradient-exploiting membrane proteins are only present on the acid face of the cell. Proton loss from inside the cell therefore depends on the rate of influx of OH^−^ from alkaline fluids, which neutralize protons within the cell, and the rate of loss of protons across the lipid phase to the alkaline exterior (Figure 2). We also consider membrane permeability to Na^+^, K^+^, and Cl^−^ ions, which move charge, and hence influence the electrochemical potential difference and the rate of proton flux. By calculating the overall proton flux on the basis of these parameters, we estimate changes in the steady-state proton concentration inside the cell relative to the outside, giving the free energy (−ΔG) available to drive carbon and energy metabolism. Our findings allow us to propose a new and tightly constrained bioenergetic route map leading from a leaky LUCA dependent on natural proton gradients, to the first archaea and bacteria with highly distinct ion-tight phospholipid membranes. These bioenergetic considerations give striking insights into the nature of LUCA, and the deep divergence between archaea and bacteria.

## Results

### Free-Energy Availability Depends on Membrane Permeability

**Figure 3:**
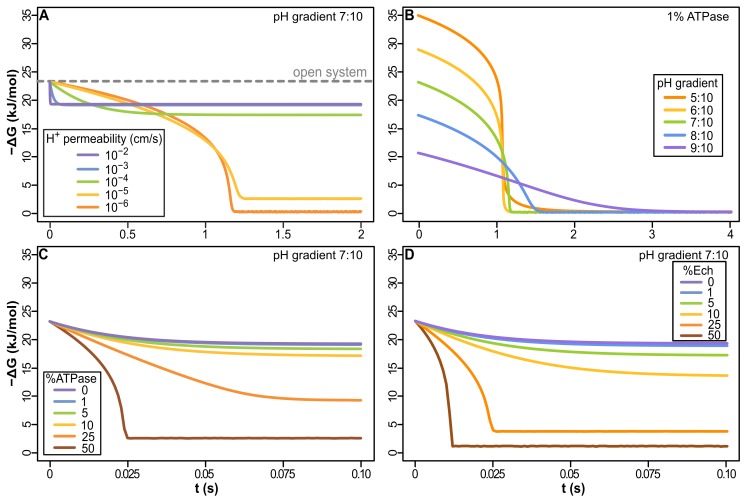
Dynamics of free-energy change (−ΔG) in cells powered by natural proton gradients. (A) Proton-permeable vesicles (≥10^−4^ cm/s) have only a small loss of free-energy compared with an open system (pH gradient 7∶10, 1% ATPase). Reduced membrane permeability (≤10^−4^ cm/s), including permeabilities equivalent to modern membranes (<10^−5^ cm/s), collapse the gradient within seconds. (B) At low permeability (10^−6^ cm/s), −ΔG collapses regardless of gradient size. Within seconds, H^+^ flux through ATPase equilibrates with the acidic fluids. (C) The collapse of −ΔG is more extensive the greater the amount of membrane-bound ATPase, even with a leaky membrane (10^−3^ cm/s). (D) With Ech, the collapse of the natural gradient is similar to that of the ATPase, showing that natural proton gradients can power energy (ATPase) and carbon (Ech) metabolism, given 1%–5% enzyme in membrane. Na^+^ permeability was kept 6 orders of magnitude higher than that of H^+^ throughout all simulations in this and all figures of the article. Except in (B), all results were calculated in a pH gradient 7∶10.

The model shows that cells with 1% ATPase in a proton-tight membrane with glycerol-phosphate headgroups (giving an H^+^ permeability <10^−5^ cm/s, like extant archaea and bacteria [34]), collapse natural proton gradients within seconds (Figure 3A and 3B). The magnitude of the pH gradient depends on the environmental setting. To constrain possibilities we considered pH values commensurate with alkaline hydrothermal vents, but the same principles apply to any other setting with dynamic pH gradients across short distances. The early oceans may have been mildly acidic, as low as pH 5, and alkaline fluids as high as pH 11 [35] but we conservatively set a 3 pH-unit gradient, with the “acid” at pH 7 and alkaline fluids at pH 10. Nonetheless, collapse of the gradient was evident in proton-tight membranes across a range of gradients (Figure 3B). Protons enter through the ATPase faster than they can exit or be neutralized by OH^−^, so H^+^ influx rapidly reaches electrochemical equilibrium. In contrast, leaky protocells (equivalent to fatty-acid vesicles without glycerol phosphate headgroups) in a 7∶10 pH gradient with 1% ATPase in the membrane retain nearly all the free energy available, having a −ΔG only ∼17% lower than an open system (i.e., a single membrane containing the same number of membrane proteins, separating a continuous flux of acid and alkaline fluids; Figure 3A). This is because proton flux through the ATPase is ∼4 orders of magnitude faster than through the lipid phase, even with a high proton permeability of 10^−2^ cm/s (based on the kinetics of proton-flux through the ATPase, see Materials and Methods and Table S1). Leaky cells in natural proton gradients of 3 pH units therefore have sufficient free energy to drive ATP synthesis.

Even leaky cells are sensitive to the amount of membrane protein, with higher proportions of ATPase collapsing the gradient (Figure 3C). In this case, the rate of H^+^ entry through ATPase covering 10%–50% of the membrane surface area is substantially faster than the rate of clearance of H^+^ from inside the cell (and reaction with OH^−^), collapsing −ΔG. However, 1%–5% ATPase in a leaky membrane (10^−3^ cm/s) retains a −ΔG of close to 20 kJ/mol (Figure 3A and 3C). With 3–4 protons translocated per ATP synthesized (Table S1), this gives a −ΔG for ATP hydrolysis of 60 to 80 kJ/mol, similar to modern cells and sufficient to drive intermediary biochemistry, including aminoacyl adenylation in protein synthesis [36]. This assumes the same stoichiometry as the modern ATPase (3–4 protons per ATP). Because the kinetics of early enzymes would arguably not have been as honed by evolution as their modern equivalents, we used 10% of modern proton flux rates. However, this difference in efficiency actually has limited impact on the model compared with modern flux rates (Figure S1); increasing the stoichiometry of the ATPase has a similarly small effect (Figure S2). We did not estimate rates of ATP synthesis, as that would require additional assumptions about concentrations of ATP, ADP, and phosphate, as well as the rates of ATP consumption and growth; these are almost impossible to constrain at present.

The same principles apply to carbon metabolism. We consider whether the membrane protein Ech could drive carbon reduction by H_2_ in natural proton gradients. Ech uses the proton-motive force to drive carbon metabolism in some archaea and bacteria via the reduction of ferredoxin [26]. As with the ATPase, cells with 1%–5% Ech in the membrane retain most of the free energy available from a 7∶10 pH gradient (Figure 3D). Higher concentrations of Ech (10%–50%) collapse −ΔG even more than the ATPase, as the rate of proton flux through Ech is double that of the ATPase, and its surface area is slightly smaller, so there are more proton pores per unit surface area (Table S1). Such high concentrations of Ech or ATPase are in any case improbable, and not relevant to modern cells, but demonstrate the range of conditions in which natural gradients can in principle drive carbon and energy metabolism.

Given a 7∶10 pH gradient, it is therefore feasible to have 1%–5% Ech and 1%–5% ATPase in the membrane, driving both carbon and energy metabolism in cells with leaky membranes. But incorporation of either G1P or G3P glycerol-phosphate headgroups (found in archaea and bacteria respectively), or racemic mixtures of archaeal and bacterial lipids (which, surprisingly, are as impermeable to protons as standard membranes [37]), are not favored because they reduce the proton permeability of the membrane and so collapse the energetic driving force. Glycerol-phosphate headgroups in particular decrease proton permeability, as they prevent fatty acid flip-flop across the membrane (see Discussion).

### Pumping Ions across Leaky Membranes Does Not Give a Sustained Increase in Free Energy

If leaky cells with low amounts of ATPase and Ech (1%–5%) are viable in natural proton gradients, but cells with phospholipid membranes are not, then the evolution of active pumping becomes a paradox: pumping protons across a proton-permeable membrane does not increase free energy (−ΔG), because the protons immediately return through the lipid phase of the membrane.

**Figure 4:**
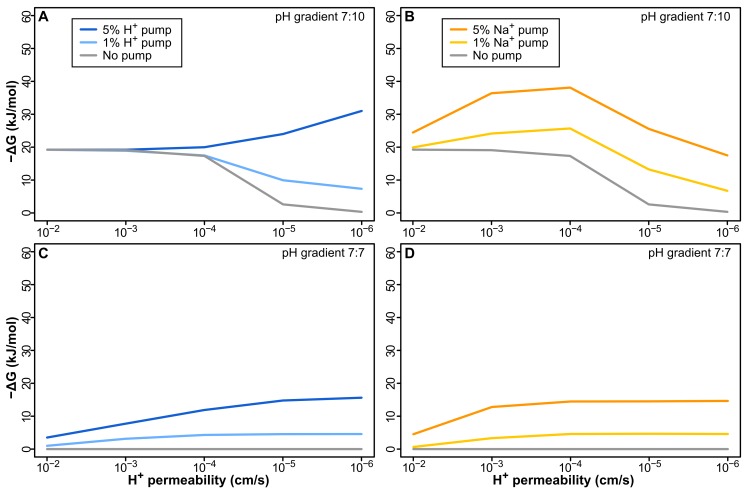
Pumping H^+^ or Na^+^ does not offer a sustained selective advantage. (A) Pumping H^+^ in a membrane with 1% ATPase causes a sustained loss in −ΔG as membrane permeability decreases with 1% pump. Even with 5% pump, −ΔG does not change over 3 orders of magnitude, and pumping only improves −ΔG near modern membrane permeability (≤10^−5^ cm/s). (B) Pumping less-permeable Na^+^ is initially better, adding to the natural gradient, but the early benefit is lost as membranes become tighter, due to the collapse of the natural H^+^ gradient. In the absence of a gradient, pumping both H^+^ (C) and Na^+^ (D) offers a sustained advantage to tightening up membranes, but given a minimal requirement of around 15–20 kJ/mol to power aminoacyl adenylation, the energy attained is not sufficient to power intermediary biochemistry.

We demonstrate this using a model of a simple H_2_-dependent proton pump (equivalent to Ech operating in reverse, as found in some simple bacteria and archaea [26]). We find that in a 7∶10 pH gradient −ΔG falls as membrane permeability decreases from 10^−2^ to 10^−6^ cm/s (Figure 4A). −ΔG here depends on two factors: active pumping and the natural pH gradient. As membrane permeability falls, the contribution of the natural pH gradient also falls, undermining −ΔG. In contrast, the benefit of pumping increases, as fewer protons return through the lipid phase. The balance between these two factors depends on the strength of pumping (which equates to the number of pumps, i.e., % surface area). However, even when the pump occupies 5% of the membrane surface area, pumping H^+^ gives no advantage until a modern permeability of 10^−5^ cm/s, i.e., there is no benefit to improving permeability across 1,000-fold (Figure 4A). Thus, there is no selective pressure to drive either the origin of pumping or the evolution of modern proton-tight membrane lipids in natural proton gradients.

Pumping Na^+^ works better across leaky membranes (Figure 4B), as lipid membranes are ∼6 orders of magnitude less permeable to Na^+^ than to H^+^ (due to fatty acid flip-flop; see Discussion) [34]. However, as with pumping H^+^, −ΔG falls as the membrane becomes less permeable, because the contribution of the natural gradient also declines, giving no continuous selective advantage to pumping Na^+^. With a proton permeability <10^−5^ cm/s, there is no advantage to pumping Na^+^ at a pump density of 1%–5% surface area compared with leaky protocells lacking a pump. Pumping Na^+^ therefore offers an initial advantage, but there is no sustained selection pressure for tightening membrane permeability to modern values.

Neither is there any advantage in the absence of a natural pH gradient. This would apply to the evolution of chemiosmotic coupling in any setting that lacks natural gradients. Under this condition, pumping either H^+^ (Figure 4C) or Na^+^ (Figure 4D) offers a steadily amplifying advantage as membrane permeability falls. However, without an external pH gradient, −ΔG is low, the rise with reduced permeability is meager, and remains well below the 15–20 kJ/mol required by modern cells to drive processes like aminoacyl adenylation for protein synthesis [36]. Cells with permeable membranes (10^−2^–10^−4^ cm/s) are therefore unlikely to be viable unless powered by some other means [23],[27]. Hence in either the presence or absence of pH gradients, there is no sustained selection pressure to drive the evolution of either active pumping or modern membranes.

### Promiscuous H^+^/Na^+^ Bioenergetics Facilitates Spread and Is Prerequisite for Active Pumping

Our model shows that leaky membranes were necessary to survive in natural proton gradients but that pumping protons across such leaky membranes is fruitless. Yet free-living cells require ion-tight membranes and active pumping for bioenergetics. What drove this evolutionary change?

We hypothesize that a necessary first step was adding Na^+^ as an additional “promiscuous” coupling ion. A non-electrogenic sodium-proton (1Na^+^/1H^+^) antiporter (SPAP), found widely in cells, could in principle use a natural H^+^ gradient to generate a biochemical Na^+^ gradient. Exchanging Na^+^ for H^+^ does not alter membrane potential directly, but the difference in lipid permeability of the two ions alters ion flux, with significant effects on −ΔG. Because lipid membranes are ∼6 orders of magnitude less permeable to Na^+^ than to H^+^ [34], fewer Na^+^ ions can pass through the lipid phase of the membrane, so the Na^+^ gradient does not dissipate as quickly. As a result, Na^+^ flux becomes more tightly funneled through membrane proteins, improving the coupling of the membrane without changing its chemistry [19]. Because the H^+^ gradient is sustained geochemically, SPAP simply adds a Na^+^ gradient to the natural H^+^ gradient. Taking advantage of mixed Na^+^/H^+^ gradients requires promiscuity of membrane proteins for both ions, which is indeed the case for several contemporary bioenergetic proteins, including the ATPase [38] and Ech [26] (see Discussion).

**Figure 5:**
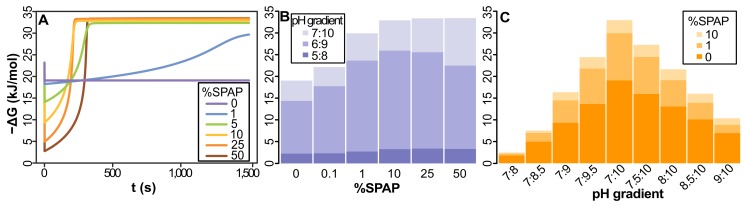
SPAP significantly increases free energy. (A) Because external Na^+^ concentration (0.4 M) is higher than H^+^ concentration (10^−7^ M), SPAP initially collapses −ΔG, and it takes minutes for the 1∶1 H^+^∶Na^+^ exchange to increase −ΔG; eventually it renders an increase of ∼60%. (B) The greatest increases are attained in relatively alkaline pH 7∶10 environments, saturating as % surface area rises. Despite equivalent gradient sizes, the absolute difference in H^+^ and OH^−^ concentrations means a 6∶9 gradient gives a lower −ΔG, as the rate of H^+^ influx is greater while neutralizing OH^−^ influx is lower. A 5∶8 gradient undermines −ΔG further, with or without SPAP. (C) SPAP facilitates colonization of environments with weaker proton gradients. 1% SPAP pushes −ΔG above 20 kJ/mol in a 7.5∶10 gradient, whereas 10% SPAP salvages an otherwise unviable 8∶10 gradient. All simulations with 1% promiscuous ATPase, no pump, no Ech, and H^+^ permeability 10^−3^ cm/s.

SPAP increases proton influx, initially lowering −ΔG (Figure 5A). However, the coupled extrusion of relatively impermeable Na^+^ ions increases −ΔG by ∼60% within minutes in a 7∶10 gradient, saturating when SPAP covers ∼5% of the membrane surface area (Figure 5A). Importantly, the free energy available from pH gradients declines in more acidic conditions. −ΔG is greatest with a 7∶10 gradient, lower at 6∶9, and nearly zero with a 5∶8 gradient, despite the three-order-of-magnitude correspondence (Figure 5B). This asymmetry arises because H^+^ and OH^−^ flux through the membrane depends on concentrations as well as gradient size [39]. Comparatively high acidity and low alkalinity increases H^+^ influx but hinders OH^−^ neutralization, collapsing the H^+^ gradient. Because Na^+^ extrusion through SPAP depends on the natural H^+^ gradient, SPAP increases −ΔG in relatively alkaline regions (pH 7–10 and 6–9) but has little effect on −ΔG in more acidic regions (pH 5–8), making acidic regions less favorable for colonization, even with SPAP. When the rate of H^+^ influx does not collapse the proton gradient, SPAP significantly increases −ΔG, allowing survival in shallower pH gradients (Figure 5C). If a −ΔG>15 kJ/mol is needed for growth, 5%–10% SPAP allows cells to grow in 50-fold weaker gradients (e.g., 8.5∶10; Figure 5C), a significant ecological advantage, facilitating spread. This general principle holds whatever the actual value of −ΔG needed for growth in early cells. The advantage offered by SPAP also applies to fluctuations in gradient size (e.g., due to mixing of fluids). −ΔG plainly fluctuates with the pH front even in the presence of SPAP; but SPAP still increases −ΔG even with considerable fluctuations in pH (Figures S3 and S4).

**Figure 6:**
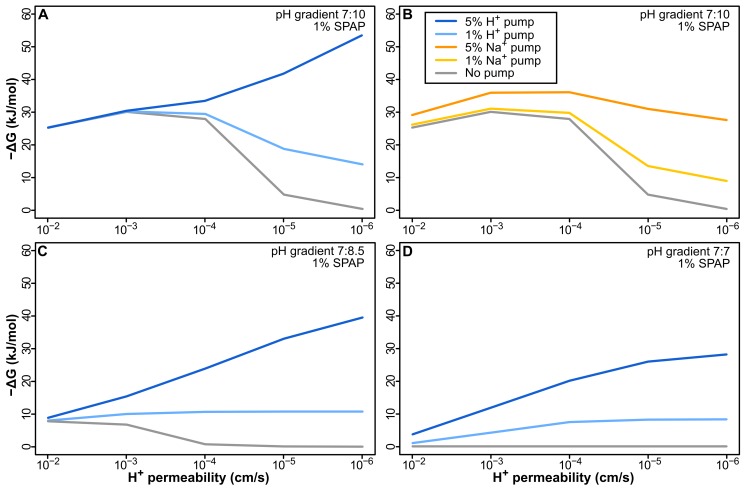
SPAP gives a sustained benefit to pumping favoring tighter membranes and allowing free living. (A) The combination of SPAP with 5% H^+^ pump gives a sustained increase in −ΔG as membrane permeability decreases, for the first time favoring the evolution of modern proton-tight phospholipid membranes. In contrast, 1% H^+^ pump gives an initial benefit, but provides insufficient power to sustain −ΔG as the gradient is lost with decreasing permeability. (B) The combination of SPAP with both 1% and 5% Na^+^ pump provides an initial benefit, but neither provides enough power to sustain −ΔG with decreasing permeability. (C) SPAP facilitates colonization of smaller gradients, ultimately making it possible to survive, after the evolution of tight membranes, in the total absence of a gradient (D); cells could not survive without a gradient unless relatively proton-tight membranes were already in place, as −ΔG falls well below the 15–20 kJ/mol threshold upon losing the gradient with a leaky membrane. All simulations assume 1% SPAP. Legend in (B) is common to all panels.

Crucially, SPAP is also a necessary preadaptation for the active pumping of protons, and for decreasing membrane permeability towards modern values. Whereas pumping H^+^ in the absence of SPAP gives no sustained benefit in terms of −ΔG, the presence of SPAP in a leaky membrane allows pumping of H^+^ to pay dividends. −ΔG now markedly increases with decreasing permeability (Figure 6A), for the first time giving a sustained selective advantage to higher levels of pumping and tighter membranes. As in the absence of SPAP, −ΔG depends on two factors: the power of the pump (which varies with the proportion of surface area covered) and the natural pH gradient. As membrane permeability falls, the contribution of the natural pH gradient also falls. While 1% pump cannot sustain −ΔG when the contribution of the gradient is lost, 5% H^+^ pump gives a steadily amplifying advantage to lowering membrane permeability (Figure 6A). Much the same applies to pumping Na^+^ (Figure 6B). The lower permeability of Na^+^ gives an initial benefit to pumping this ion, but this is lost as the membrane becomes tighter, even with 5% pump (Figure 6B). This lower efficacy is due to the much higher external concentration of Na^+^.

With active pumping, tighter membranes, and SPAP, cells could colonize more acidic regions (Figure S5), regions with weaker gradients (Figure 6C), and ultimately survive in the absence of a gradient altogether (Figure 6D). With no external pH gradient, SPAP interconverts efficiently between H^+^ and Na^+^, making it feasible to pump either ion (Figure 6D). These cells are now modern in that they have a fully functional chemiosmotic circuit and proton-tight membranes, and hence could evolve the traits required to leave the natural gradients for the external world. We propose that this process occurred independently in divergent populations that had spread widely using SPAP to colonize regions with weak gradients (see Discussion). These independent populations subsequently evolved into the two main branches of early life, the archaea and bacteria [1].

## Discussion

Our model suggests a resolution to the long-standing paradox that membrane bioenergetics are universal, but membranes are fundamentally different [19]. In so doing, the model gives a striking insight into the deep evolutionary split between archaea and bacteria. It reveals that the late and divergent evolution of impermeable membranes could have arisen as a simple outcome of LUCA's exploitation of natural proton gradients. Our model applies in principle to any environment in which sharp differences in proton concentration are sustained over short distances, one concrete example being alkaline hydrothermal vents [18],[24],[31]–[33]. Given the membrane proteins Ech and ATPase, we show that natural proton gradients could have sustained both carbon and energy metabolism in LUCA (Figure 3C and 3D). However, to do so, LUCA had to have very leaky membranes, the only way to avoid deadly electrochemical equilibrium (Figure 3A).

Our results indicate that LUCA did not have modern phospholipids. The addition of glycerol-phosphate headgroups is specifically precluded by the requirement for high proton-permeability in natural gradients (Figure 3A). Addition of a glycerol-phosphate headgroup reduces proton permeability substantially, as the polar headgroup cannot cross the hydrophobic interior of the membrane [40]. In contrast, lipid membranes composed of mixed amphiphiles, including fatty acids, have much greater proton permeability, through “flip-flop.” In flip-flop, protonation of a negatively charged fatty acid eliminates its charge, allowing the neutral residue to migrate across the hydrophobic membrane to the inside [41]. Deprotonation on the relatively alkaline interior rapidly dissipates proton gradients, explaining the high proton permeability of fatty acid vesicles [41]. Flip-flop is not possible with Na^+^, which remains ionic in the presence of a negatively charged amphiphile, hence its lower permeability [34]. Our results indicate that LUCA was sophisticated in terms of genes and proteins, but did not have a modern phospholipid membrane. However, LUCA must have had a stable lipid bilayer membrane composed of mixed amphiphiles, probably including fatty acids and isoprenes (some of which are found in both archaea and bacteria [15]). A lipid bilayer membrane is undoubtedly necessary for the function of membrane proteins such as the ATPase and Ech [42].

The actual permeability of membranes is difficult to determine experimentally, as H^+^ permeability depends in part on the permeability of counter-ions, and therefore varies with the composition of solutions used in measurements. Values of phospholipid membrane H^+^ permeability range from 10^−4^ cm/s [43] to 10^−10^ cm/s [44],[45], with a consensus favoring a value of between 10^−4^ to 10^−6^ cm/s [34]. The H^+^ permeability of fatty acid vesicles is higher, in the range of 10^−2^ to 10^−3^ cm/s or even greater [41]. These values are for standard temperature, 25°C (298 K). Both H^+^ and Na^+^ permeability rise substantially with temperature, by approximately 1 order of magnitude for every 20°C increase between 20°C and 100°C, although the actual values depend on membrane composition [45]. The membrane permeability also depends on the kinetics of membrane proteins, which likewise vary with temperature. We have used standard temperature for enzyme kinetics. How these values would vary with temperature is difficult to estimate, as the kinetics of enzymes adapted to low temperatures would differ from those in thermophiles if placed in the same membrane at the same temperature. However, our simulations of efficiency and stoichiometry (Figures S1 and S2) suggest that the effect should be substantially less than that of lipid permeability. We are therefore confident that our results apply generally, despite these uncertainties. We stress that our argument relates to the principle of energy transduction in natural proton gradients, not to the specific values used for membrane permeability. The key point is that leaky membranes were essential to transduce natural proton gradients, and there was no advantage to be gained by the evolution of proton-tight phospholipid membranes, whether at low or high temperatures.

This leads to a paradox. Pumping either H^+^ or Na^+^ over leaky membranes gives no sustained advantage when membrane permeability is lowered over 1,000-fold (Figure 4A and 4B). That precludes the evolution of either active ion pumps or modern proton-tight membranes in a LUCA dependent on natural proton gradients. We hypothesize that the evolution of a SPAP was the key innovation that favored the independent evolution of active ion pumps and phospholipid membranes in bacteria and archaea. SPAP has two major effects that made this possible.

First, SPAP favors divergence, through adding a Na^+^ gradient to the geochemically sustained H^+^ gradient. Because lipid membranes are much less permeable to Na^+^ ions, these preferentially flow back through membrane proteins, thereby increasing free-energy availability by up to 60% (Figure 5A). For this additional Na^+^ gradient to be useful, membrane proteins must be promiscuous for Na^+^ and H^+^, which is the case for some primitive ATPase enzymes [38] and for Ech [26]. While the ATPase generally specializes either for H^+^ or Na^+^ today, only a few amino acid changes are required to switch from one form to the other [46]. Phylogenetic trees of the ATPase suggest that the H^+^-dependent and Na^+^-dependent forms are interleaved, implying greater promiscuity in early evolution [24]. The reason probably relates to the close similarity in ionic radius and charge of Na^+^ without its hydration shell (the form in which it usually passes through membrane proteins) and the hydronium ion, H_3_O^+^ (the form in which H^+^ is most commonly found in solution). Thus it is likely that addition of a Na^+^ gradient to a natural H^+^ gradient by SPAP would indeed increase the free energy available to the cell as a usable electrochemical difference. This enabled cells to survive in 50-fold lower gradients (Figure 5C), or with intermittent gradients and mixing (Figures S3 and S4), facilitating spread and divergence.

Second, SPAP gives a continuous selective advantage to actively pumping protons even across a leaky membrane (Figure 6A). This advantage amplifies steadily as membrane permeability decreases, all the way towards values for largely impermeable modern membranes (Figure 6A). Our results lead us to suggest that the SPAP is ancestral and must have been present in LUCA. Phylogenetic analysis is consistent with this prediction. BLAST [47] results show a match for archaeon *Methanocaldococcus jannaschii*'s Mj1275 SPAP to an equivalent or very closely related protein in at least one member of 35 out of all 37 prokaryotic phyla reported to date (Table S2). The two bacterial clades with a missing match are to date single-member phyla whose only known species may have either lost the gene over time, had it diverge beyond observable similarity to the *M. jannaschii* ortholog, or simply have not been fully annotated in the databases yet. This confirms our prediction of the universality of SPAP in spite of the stark dissimilarity in membranes, and paves the way for closer phylogenetic analysis of these antiporters and related proteins.

We note that the early operation of SPAP would have the effect of lowering the intracellular Na^+^ concentration substantially below ambient seawater concentration, explaining how cells that evolved in the ocean could nonetheless be optimized to low intracellular Na^+^ and high K^+^ concentration. The operation of antiporters (and possibly symporters), driven by natural proton gradients, could in principle have modulated intracellular ionic composition to the low-Na^+^–high-K^+^ characteristic of most modern cells, leading to selective optimization of protein function without the need for a specific terrestrial environment with a particular ionic balance [27]. These considerations are also consistent with the universality of SPAP across prokaryotic phyla.

Our analysis demonstrates that active ion pumps almost certainly arose after SPAP, and only then did selection favor the evolution of ion-tight membranes with glycerol phosphate headgroups. Given that SPAP in itself facilitated the spread and colonization of regions with shallower (Figure 5C) or more intermittent gradients (Figures S3 and S4), pumping is expected to arise independently in more than one population, as observed [13],[19]. Only when active ion pumping had evolved was there any benefit to incorporating glycerol-phosphate headgroups, thereby reducing membrane permeability (Figure 6A). Phospholipid biosynthesis involves nucleophilic attack on the prochiral carbonyl center of dihydroxyacetone phosphate [10]. This can be achieved from either side of the molecule, giving rise to opposite stereochemistries of the central carbon in glycerol phosphate (Figure 1). The enzymes involved, G1PDH in archaea and G3PDH in bacteria appear to have taken these alternatives by chance in independent populations that had already evolved distinct ion pumps. Thus we posit that the ancestors of archaea and bacteria evolved both ion pumps and phospholipid membranes independently, the latter on the basis of a simple binary choice in the orientation of nucleophilic attack on dihydroxyacetone phosphate.

**Figure 7:**
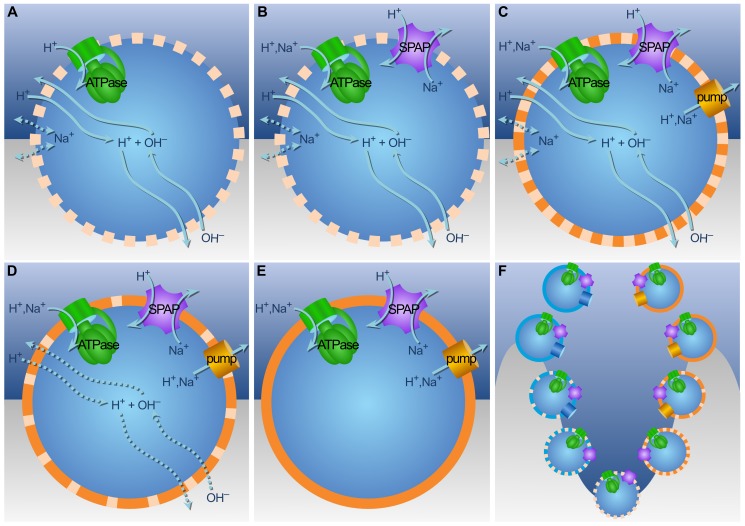
Divergence of archaea and bacteria. (A) Ions cross the membrane in response to concentration gradients and electrical potential. OH^−^ neutralizes incoming protons. The H^+^ gradient can drive energy metabolism via ATPase, and carbon metabolism via Ech (not shown). (B) SPAP generates a Na^+^ gradient from the H^+^ gradient. As Na^+^ is less permeable than H^+^, SPAP improves coupling, given promiscuity of membrane proteins for H^+^ and Na^+^. (C) Membrane pumps generate gradients by extruding H^+^ or Na^+^ ions. (D) Exploiting natural gradients demands high membrane permeability, but pumping with SPAP drives the evolution of tighter membranes, facilitating colonization of less alkaline environments. (E) Impermeable membranes funnel ion flow through bioenergetic proteins, independent of natural gradients. (F) From bottom up, SPAP favors divergence, selection for active pumping and tighter membranes. Pumping and phospholipid membranes arose independently in archaea and bacteria.

We conclude that the membranes of LUCA were necessarily leaky, composed of mixed amphiphiles (including fatty acids) but lacking glycerol-phosphate headgroups. Fatty-acid vesicles have long been considered plausible protocells because of their simplicity, stability, and dynamic ability to grow [48]–[50], but are generally thought unsuitable for chemiosmotic coupling due to their high proton permeability [27],[51]. Leaky membranes have therefore generally been interpreted in terms of heterotrophic origins of life [52]. In contrast, we find that high proton permeability was in fact indispensable to drive both carbon and energy metabolism in natural proton gradients, consistent with autotrophic origins; and this requirement for leaky membranes in turn precluded the early evolution of phospholipid membranes (Figure 7). Our model offers a selective basis for the universality of membrane bioenergetics and the ATPase, while elucidating the paradoxical differences in membranes and active ion pumps. The deep disparity between archaea and bacteria in carbon and energy metabolism [19],[53], and in membrane lipid stereochemistry [10], reflects two independent origins of active pumping in divergent populations (Figure 7).

The conclusion that LUCA had leaky membranes, and that modern phospholipid membranes evolved later and independently in archaea and bacteria, provides a framework for interpreting other dichotomies between archaea and bacteria. The late and independent evolution of glycolysis but not gluconeogenesis [12] is entirely consistent with LUCA being powered by natural proton gradients across leaky membranes. Several discordant traits are likely to be linked to the late evolution of cell membranes, notably the cell wall, whose synthesis depends on the membrane [11] and DNA replication [14]. In the latter case, the fingers-thumb-palm motif at the active site of DNA polymerase enzymes [54] and the structure of the replication fork [55] are superficially similar in archaea and bacteria, yet most proteins involved in DNA replication, including the principal replicative polymerases, bear no phylogenetic resemblance [14],[56],[57]. That implies either independent origins [14] or inscrutably deep divergence compared with the plainly homologous transcription and translation machinery [56],[57]. Because the bacterial replicon is attached to the plasma membrane during cell division [58]–[60], this complex presumably arose after (or coevolved with) the bacterial membrane, which must have driven a deep phylogenetic disparity, even if DNA replication had arisen in LUCA. Thus key facets of the fundamental split between archaea and bacteria could be linked to the late origin of phospholipid membranes, for these bioenergetic reasons. While it is difficult to prove that these bioenergetic factors really did account for the deepest branch in the tree of life, they do offer a robust and testable framework that can explain the paradoxical character of LUCA and the stark differences between archaea and bacteria.

## Materials and Methods

### General Description of the Model

Cells were modeled half embedded in the alkaline fluid, with the other half exposed to the comparatively acidic fluid. This produced an inward proton gradient from the acidic side, sustained by the constant neutralization with OH^−^ from the alkaline side (Figure 2). Only the two external pH values are fixed; the internal pH is then arrived at in response to the fluxes of H^+^ and OH^−^ across the membrane, which in turn depends on permeability, the respective concentrations of each ion, and flow through the membrane proteins. Equation 1 describes the various ways in which protons could enter or leave the cell at every time step: by simple diffusion across the membrane on either side, and through any of the membrane proteins, namely the ATPase, SPAP, pump, or Ech.
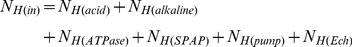
(1)Total concentrations of H^+^ and OH^−^ were calculated at every time step by neutralization and equilibration to the dissociation constant of water. External fluids were assumed to be part of comparatively large bodies of water, with their acidity and alkalinity sustained by large-scale geological or meteorological processes; thus their concentrations of H^+^, OH^−^, and other ions were assumed constant. Analogous equations were used for other ions.

Table S1 describes the parameters chosen for the results presented in the text, unless otherwise stated.

We anticipate that enzymes could not have reached their current reaction rate values at the early stages of evolution that we are considering, so for the results presented in the main text we have consistently used 10% of the current turnover rates referenced in Table S1. A series of results using modern (100%) turnover rates are presented in Figure S1 for comparison.

### Flux through the Membrane

Membrane flux J_S_ of a neutral substance S was modeled using a traditional passive diffusion equation [61]

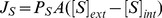
(2)where P_S_ is the permeability of the substance, A is the area of the membrane, and [S]_ext_ and [S]_int_ are the external and internal concentrations respectively. To account for the effect of membrane potential Δψ on the behavior of charged particles, ion diffusion was modeled using the Goldman-Hodgkin-Katz flux equation [39],[62]

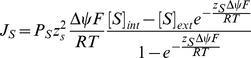
(3)where z_s_ is the charge of the substance, F and R are the Faraday and gas constants, respectively, and T is the temperature. Electrical membrane potential Δψ was in turn modeled using the Goldman-Hodgkin-Katz voltage equation [39],[62]

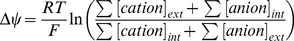
(4)for the concentration of each cation and anion present.

Internal protons and hydroxide were equilibrated using the dissociation constant of water.

### Free Energy (ΔG) Calculations

The available free energy ΔG from the H^+^ gradient was modeled with the traditional equation used by Mitchell [20]

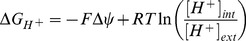
(5)An analogous equation was used for the Na^+^ gradient.

The power of ATP to catalyze biochemical reactions in the cell comes not specifically from hydrolysis of the molecule itself but from the degree to which the ATP/ADP ratio is shifted from thermodynamic equilibrium; that is, the energy available from ATP hydrolysis varies with the ATP/ADP ratio [30]. The equilibrium constant and thus the energy required for ATP synthesis depends on the concentrations of ADP, phosphate, and magnesium ion, as well as pH [20],[30], but with the exception of pH these values are unknown for the systems modeled, as are rates of ATP hydrolysis. We have therefore used Equation 5 to calculate the size of the electrochemical gradient (ΔG) as a function of the H^+^ and Na^+^ gradients and the electrical membrane potential (Δψ). The steady-state ΔG in turn gives an indication of how far from equilibrium the ATP/ADP ratio could be pushed. With 3–4 protons translocated per ATP, a steady-state ΔG of −20 kJ/mol is large enough to drive the ATP/ADP ratio to a disequilibrium of 10 orders of magnitude, equivalent to that found in modern cells [30].

We calculated steady-state ΔG as a function of the size of the H^+^ and Na^+^ gradients and the electrical membrane potential (Δψ) between the acid fluid and the inside of the cell. These factors in turn depend on steady-state rates of proton flux into and out of the cell via the lipid phase of the membrane (specified by its H^+^ and Na^+^ permeability and surface area) and through the ATPase. We calculated the maximum flux of H^+^ or Na^+^ flux through the ATPase on the basis of the maximum possible number of ions translocated per second. Maximum ion flux is based on the reported maximum turnover rate of ATPase (Table S1), i.e., the maximum number of ATP molecules that each ATPase unit can synthesize in one second when operating at top speed, multiplied by 3.3, the number of H^+^ or Na^+^ required to synthesize 1 ATP (Table S1). This number was then multiplied by the number of ATPase units in the system, estimated from the membrane surface area assigned to this protein in each simulation (e.g., 1%, 5%, etc.) and the reported surface area of the membrane-integral F_O_ subunit (Table S1).

We further assumed that the actual flux rate of H^+^ and Na^+^ through the ATPase would also depend on the driving force itself, ΔG, i.e., the size of the H^+^/Na^+^ gradient and the electrical membrane potential (Δψ). We assumed that the ATPase obeys hyperbolic Michaelis-Menten dynamics, commonly the case in enzyme kinetics [63] and reported for the ATPase [64], such that H^+^/Na^+^ flux asymptotically approaches the maximum turnover rate when the driving force is large, again assuming that flux rate is unconstrained by ADP availability. Thus, increasing ΔG beyond a threshold cannot increase H^+^/Na^+^ flux beyond the maximum turnover rate, so flux rate must saturate. The hyperbolic curve was modeled to reach saturation slightly beyond −20 kJ/mol, a gradient large enough to drive the ATP/ADP ratio to 10 orders of magnitude disequilibrium in modern cells [30] and equivalent to a membrane potential of around 200 mV, close to a maximum for modern lipid membranes, given the low capacitance of thin lipid membranes. This number, between zero and one, was finally multiplied by the maximum flux of H^+^ or Na^+^, described above, to determine the influx of each of the two ions through the ATPase. When added to H^+^/Na^+^ flux rates across the lipid phase, the steady-state H^+^/Na^+^ flux through the ATPase gave a steady-state ΔG available to drive ATP synthesis.

Full promiscuity of the ATPase to Na^+^ and H^+^ was assumed, with preference of one ion over the other depending solely on their respective gradient sizes. The Ech was modeled analogously.

### Modeling the Sodium-Proton Antiporter and Pump

SPAP was modeled to respond to the H^+^ and Na^+^ gradients, exchanging ions in the direction determined by the larger of the two gradients. Δψ was assumed to affect SPAP speed but not direction [65]. Since the H^+^ gradient is reversed on the alkaline side, we assumed the SPAP, ATPase, and Ech operated only on the acidic side.

The pump was modeled as a generic system able to extrude either H^+^ or Na^+^, dependent on the concentration of hydrogen gas (H_2_), and responding to the opposing gradient, thus making it easier to pump protons against an alkaline fluid, and more difficult against an acidic fluid.

### Source Code

A running example of the code can be found at http://www.ucl.ac.uk/~rmhknjl/research/membranedivergence

This code can be run directly from any typical computer with an Internet connection. Additionally, it can be downloaded and run locally (at no significant increase in speed) from http://github.com/UCL/membranedivergence

### BLAST Searches

The primary amino acid sequence of the *M. jannaschii* Mj1275 Na^+^/H^+^ antiporter (SPAP) was obtained from the NCBI protein sequence database. Mj1275 is one of three known SPAP genes in archaeon *M. jannaschii*, the other two being Mj0057 and Mj1521 [66]. The first belongs to the NapA family, while the latter two are in the NhaP family. Phylogenetic analysis was performed on these three genes as well as the two common *Escherichia coli* SPAP genes, NhaA and NhaB [67],[68], using the NCBI-BLASTp server [47] with standard parameters, filtering for each prokaryotic phylum (considering each of the proteobacteria as a separate clade). Results for Mj1275 showed the highest hit rate (Table S2), possibly hinting that it is closest to the ancestral form of the SPAP. Results for the other genes are not shown.

## Supporting Information

Figure S1 **Comparison of different enzyme turnover rates.** We assume that membrane proteins in LUCA had lower turnover rates than those in modern archaea and bacteria. For all the results in the main text, turnover rates were modeled at 10% of modern values (see Table S1 for these values). The figure shows that with ATPase, SPAP, and pump, the behavior is similar when turnover is set at 10%, 50%, and 100% for each protein. Parameters: 5% pump, 1% ATPase, 1% SPAP, pH gradient 7∶10.
(TIF)

Figure S2 **Effect of higher H^+^-to-ATP stoichiometry in the ATPase.** Lowering the efficiency of the ATPase by increasing the number of H^+^ necessary to synthesize one ATP molecule has a minor effect on the simulation results. Almost halving efficiency to 6 H^+^ per ATP lowers −ΔG by less than 1%.
(TIF)

Figure S3 **Effect of fluctuations in external acidic pH, while holding external alkaline pH constant at pH 10.** We considered the effect of mixing, with alkaline fluids causing local fluctuations in the pH of the acidic side. These were taken to occur on a scale of seconds, causing meaningful perturbations to the pH gradient and −ΔG. (A) Increases in the pH of the acidic side shrink the exploitable gradient. (B) With 1% ATPase and no SPAP or pump in the membrane, pH fluctuations are followed swiftly by corresponding changes in −ΔG. Circles on the y axis show the −ΔG values at stasis at pH_acidic_ 7. Histograms in (C) show the frequency distributions for the corresponding curves in (B), with the vertical lines denoting the values for stasis at pH 7 (solid black) and mean of the corresponding curve (dashed grey). (D) Although responses are somewhat slower, addition of 5% SPAP makes fluctuations more survivable by increasing power overall. (E) is analogous to (C). See Figure S4 for similar fluctuations in the alkaline side.
(TIF)

Figure S4 **Effect of fluctuations in external alkaline pH, while holding external acidic pH constant at pH 7.** Qualitatively similar behavior to that of Figure S3 was observed when fluctuations occur on the alkaline side.
(TIF)

Figure S5 **Pumping in the presence of SPAP facilitates adaptation to more acidic regions.** All three curves show a steady increase in −ΔG with 5% pump in equivalent pH gradients (each of 3 pH units) with decreasing membrane permeability. In relatively alkaline conditions (pH 7∶10 and 6∶9) the benefit of pumping increases with decreasing permeability, but is relatively modest. In more acidic environments (pH 5∶8) there is initially a relatively greater payback to pumping as membrane permeability decreases. The reason is that at high membrane permeability (10^−2^ cm/s) and relatively acidic pH (5∶8), there is a fast influx of H^+^ (from the acidic side) and a slow influx of OH^−^ (from the alkaline side), leading to the collapse of −ΔG. Pumping across a very leaky membrane gives little benefit even with SPAP (−ΔG is very low). Lowering membrane permeability limits H^+^ influx and enhances the benefits of pumping, giving a greater relative benefit in acidic conditions (pH 5∶8). In contrast, with tight membranes (10^−6^ cm/s), cells are powered almost exclusively by their own pumps, with little contribution from the external gradient (−ΔG collapses in the absence of a pump; see Figure 3A and 3B). Cells in relatively alkaline (6∶9 and 7∶10) environments now gain slightly more from pumping. The reason is that the opposing external H^+^ concentration is greater at pH 5∶8, so pumping H^+^ out is harder than at pH 6∶9 or 7∶10. The figure thus shows a transition from a highly permeable gradient-powered system on the left to a low permeability pump-powered system on the right.
(TIF)

Table S1 **Parameters in the model and references.**
(DOC)

Table S2 **BLAST-search results for matches of the archaeal** ***M. jannaschii*** **Mj1275 SPAP to at least one member of each of the 37 known prokaryotic phyla.**
(DOC)

## Abbreviations

Ech: energy-converting hydrogenase
LUCA: last universal common ancestor
SPAP: sodium-proton antiporter
ATPase: ATP synthase
