# Correction: A Bioenergetic Basis for Membrane Divergence in Archaea and Bacteria

**DOI:** 10.1371/journal.pbio.1002102

**Published:** 2015-03-20

**Authors:** The PLOS Biology Staff

There is a typo in Equation 4 of the paper, which is missing the permeability (P) factor. The equation was correct in the code and this typo did not affect the results. However, after noticing this, the authors also discovered a small error in the code, which has now been corrected. This correction does not change the text or conclusions of the paper, though it does have a slight impact on the main and supporting figures. The authors have provided a corrected version of Figs. 1–7, S1–S5 Fig. and the manuscript (S1 Manuscript) here.

**Fig 1:**
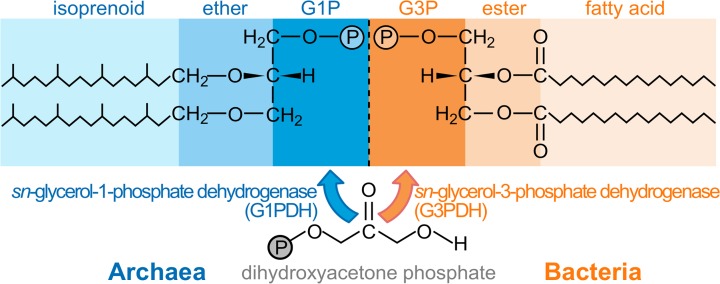
Membrane lipids of archaea and bacteria. Archaeal lipids (left) are typically composed of isoprenoid chains linked by ether bonds to an sn-glycerol-1-phosphate (G1P) backbone. The chirality of the two glycerol backbones is fully conserved within each clade not only in structure but in their unrelated synthetic enzymes. Although ether linkages have been observed in bacterial membranes [15] and isoprenoids are common to all three domains, bacterial lipids (right) are typically composed of fatty acids in ester linkage to an sn-glycerol-3-phosphate (G3P) skeleton. Despite widespread horizontal gene transfer, no bacterium has been observed with the archaeal enantiomer, or vice versa [10].

**Fig 2:**
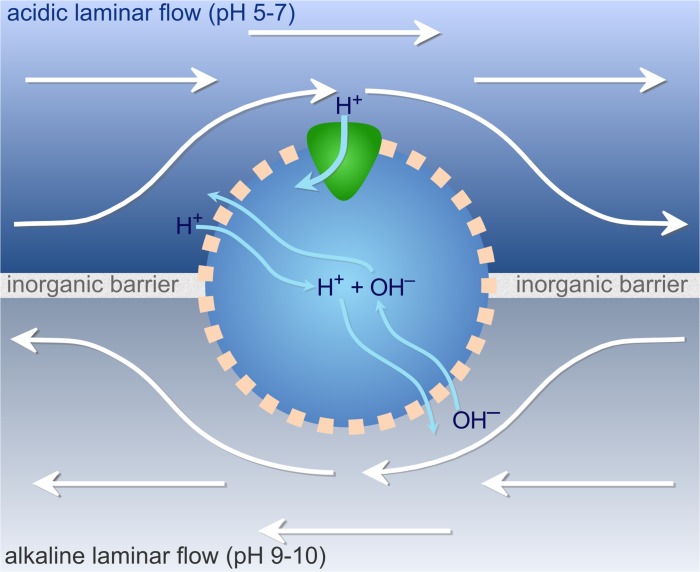
The model. A cell with a semi-permeable membrane sits at the interface between an alkaline and an acidic fluid. The fluids are continuously replenished and otherwise separated by an inorganic barrier. Hydroxide ions (OH^−^) can flow into the cell from the alkaline side by simple diffusion across the membrane, with protons (H^+^) entering in a similar manner from the acidic side. Other ions (Na^+^, K^+^, Cl^−^, not shown) diffuse similarly, as a function of their permeability, charge, and respective internal and external concentrations on each side. Inside the protocell, H^+^ and OH^−^ can neutralize into water, or leave towards either side. Internal pH thus depends on the water equilibrium and relative influxes of each ion. A protein capable of exploiting the natural proton gradient sits on the acidic side, allowing energy assimilation via ATP production, or carbon assimilation via CO_2_ fixation.

**Fig 3:**
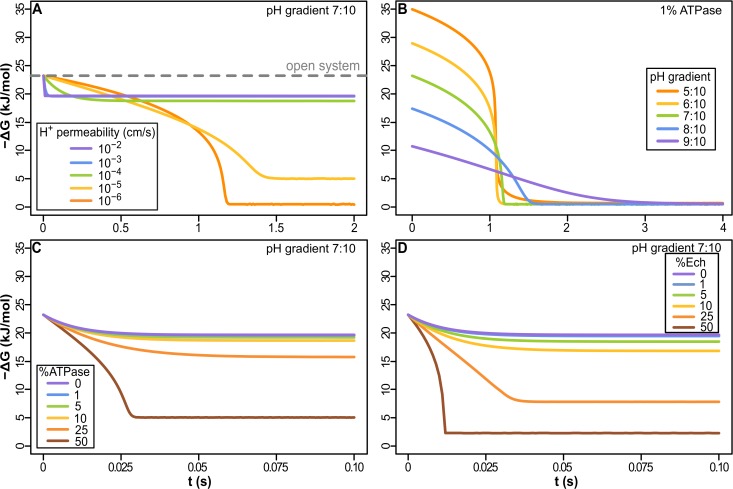
Dynamics of free-energy change (−ΔG) in cells powered by natural proton gradients. (A) Proton-permeable vesicles (≥10^−4^ cm/s) have only a small loss of free energy compared with an open system (pH gradient 7:10, 1% ATPase). Reduced membrane permeability (<10^−4^ cm/s), including permeabilities equivalent to modern membranes (≤10^−5^ cm/s), collapse the gradient within seconds. (B) At low permeability (10^–6^ cm/s), −ΔG collapses regardless of gradient size. Within seconds, H^+^ flux through ATPase equilibrates with the acidic fluids. (C) The collapse of −ΔG is more extensive the greater the amount of membrane-bound ATPase, even with a leaky membrane (10^−3^ cm/s). (D) With Ech, the collapse of the natural gradient is similar to that of the ATPase, showing that natural proton gradients can power energy (ATPase) and carbon (Ech) metabolism, given 1%–5% enzyme in membrane. Na^+^ permeability was kept 6 orders of magnitude higher than that of H+ throughout all simulations in this and all figures of the article. Except in (B), all results were calculated in a pH gradient 7:10.

**Fig 4:**
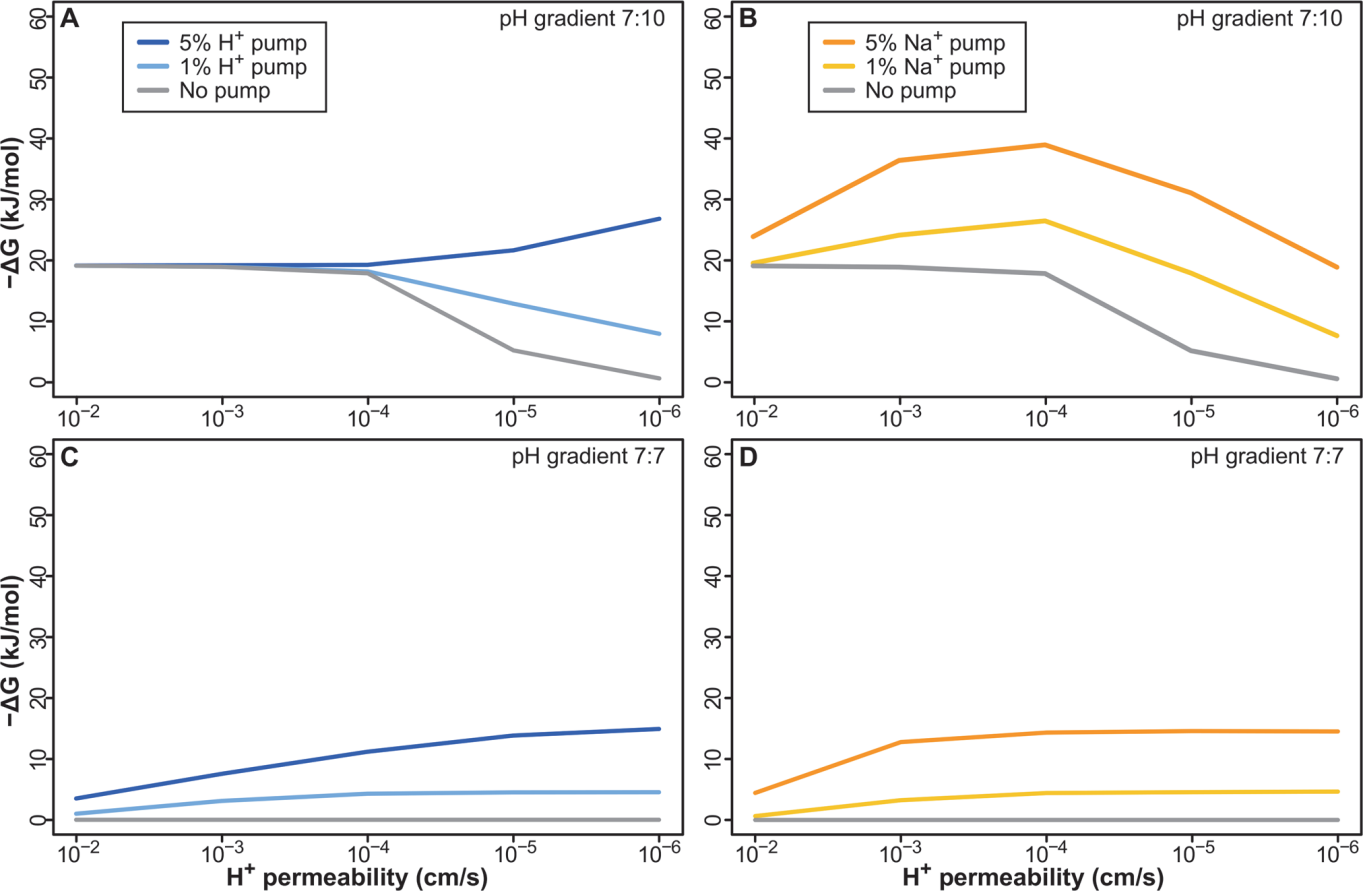
Pumping H^+^ or Na^+^ does not offer a sustained selective advantage. (A) Pumping H^+^ in a membrane with 1% ATPase causes a sustained loss in −ΔG as membrane permeability decreases with 1% pump. Even with 5% pump, −ΔG does not change over 3 orders of magnitude, and pumping only improves −ΔG near modern membrane permeability (≤10^−5^ cm/s). (B) Pumping less-permeable Na^+^ is initially better, adding to the natural gradient, but the early benefit is lost as membranes become tighter, due to the collapse of the natural H^+^ gradient. In the absence of a gradient, pumping both H^+^ (C) and Na^+^ (D) offers a sustained advantage to tightening up membranes, but given a minimal requirement of around 15–20 kJ/mol to power aminoacyl adenylation, the energy attained is not sufficient to power intermediary biochemistry.

**Fig 5:**
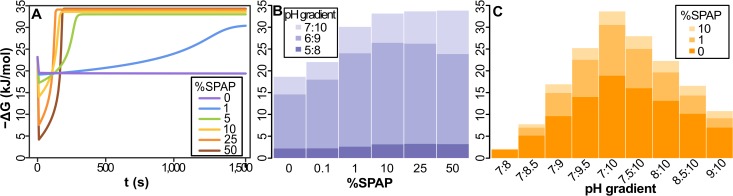
SPAP significantly increases free energy. (A) Because external Na^+^ concentration (0.4 M) is higher than H^+^ concentration (10^−7^ M), SPAP initially collapses −ΔG, and it takes minutes for the 1∶1 H^+^∶Na^+^ exchange to increase −ΔG; eventually it renders an increase of ∼60%. (B) The greatest increases are attained in relatively alkaline pH 7∶10 environments, saturating as % surface area rises. Despite equivalent gradient sizes, the absolute difference in H+ and OH− concentrations means a 6∶9 gradient gives a lower −ΔG, as the rate of H^+^ influx is greater while neutralizing OH^−^ influx is lower. A 5∶8 gradient undermines −ΔG further, with or without SPAP. (C) SPAP facilitates colonization of environments with weaker proton gradients. 1% SPAP pushes −ΔG above 20 kJ/mol in a 7.5∶10 gradient, whereas 10% SPAP salvages an otherwise unviable 8∶10 gradient. All simulations with 1% promiscuous ATPase, no pump, no Ech, and H^+^ permeability 10^−3^ cm/s.

**Fig 6:**
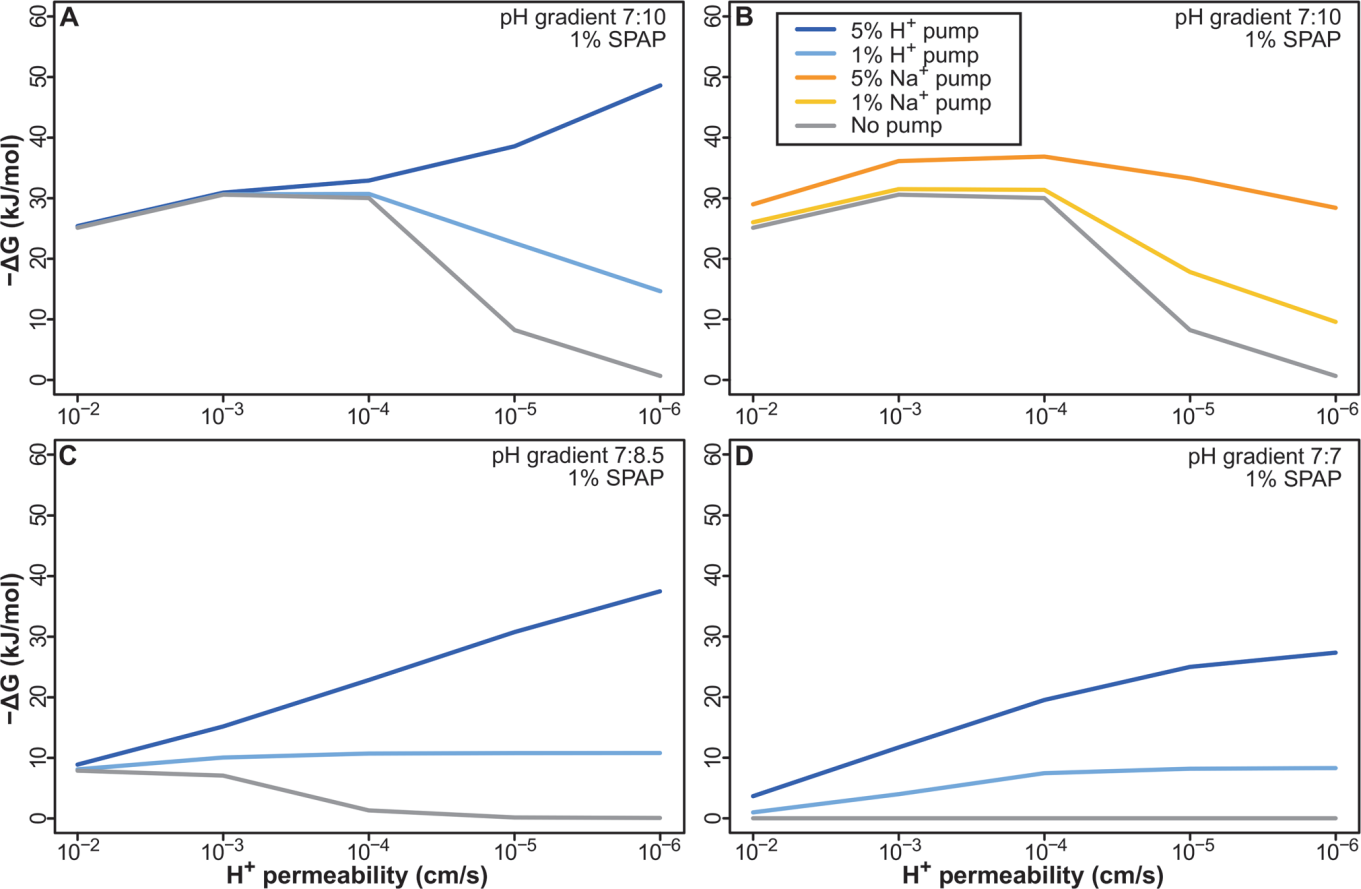
SPAP gives a sustained benefit to pumping favoring tighter membranes and allowing free living. (A) The combination of SPAP with 5% H^+^ pump gives a sustained increase in −ΔG as membrane permeability decreases, for the first time favoring the evolution of modern proton-tight phospholipid membranes. In contrast, 1% H^+^ pump gives an initial benefit, but provides insufficient power to sustain −ΔG as the gradient is lost with decreasing permeability. (B) The combination of SPAP with both 1% and 5% Na^+^ pump provides an initial benefit, but neither provides enough power to sustain −ΔG with decreasing permeability. (C) SPAP facilitates colonization of smaller gradients, ultimately making it possible to survive, after the evolution of tight membranes, in the total absence of a gradient (D); cells could not survive without a gradient unless relatively proton-tight membranes were already in place, as −ΔG falls well below the 15–20 kJ/mol threshold upon losing the gradient with a leaky membrane. All simulations assume 1% SPAP. Legend in (B) is common to all panels.

**Fig 7:**
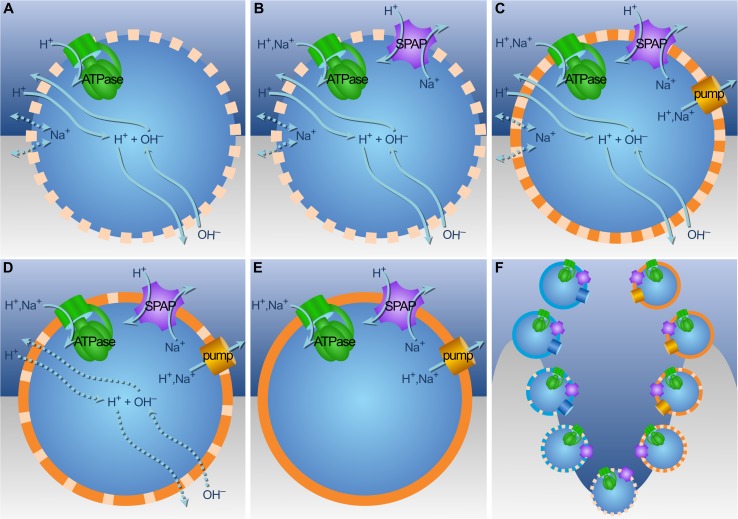
Divergence of archaea and bacteria. (A) Ions cross the membrane in response to concentration gradients and electrical potential. OH^−^ neutralizes incoming protons. The H^+^ gradient can drive energy metabolism via ATPase, and carbon metabolism via Ech (not shown). (B) SPAP generates a Na^+^ gradient from the H^+^ gradient. As Na^+^ is less permeable than H^+^, SPAP improves coupling, given promiscuity of membrane proteins for H^+^ and Na^+^. (C) Membrane pumps generate gradients by extruding H^+^ or Na^+^ ions. (D) Exploiting natural gradients demands high membrane permeability, but pumping with SPAP drives the evolution of tighter membranes, facilitating colonization of less alkaline environments. (E) Impermeable membranes funnel ion flow through bioenergetic proteins, independent of natural gradients. (F) From bottom up, SPAP favors divergence, selection for active pumping and tighter membranes. Pumping and phospholipid membranes arose independently in archaea and bacteria.

## Supporting Information

S1 Fig Comparison of different enzyme turnover rates. We assume that membrane proteins in LUCA had lower turnover rates than those in modern archaea and bacteria. For all the results in the main text, turnover rates were modeled at 10% of modern values (see S1 Table for these values). The figure shows that with ATPase, SPAP, and pump, the behavior is similar when turnover is set at 10%, 50%, and 100% for each protein. Parameters: 5% pump, 1% ATPase, 1% SPAP, pH gradient 7:10.
(TIF)

S2 Fig Effect of higher H^+^-to-ATP stoichiometry in the ATPase. Lowering the efficiency of the ATPase by increasing the number of H^+^ necessary to synthesize one ATP molecule has a minor effect on the simulation results. Almost halving efficiency to 6 H^+^ per ATP lowers −ΔG by less than 1%.
(TIF)

S3 Fig Effect of fluctuations in external acidic pH, while holding external alkaline pH constant at pH 10. We considered the effect of mixing, with alkaline fluids causing local fluctuations in the pH of the acidic side. These were taken to occur on a scale of seconds, causing meaningful perturbations to the pH gradient and −ΔG. (A) Increases in the pH of the acidic side shrink the exploitable gradient. (B) With 1% ATPase and no SPAP or pump in the membrane, pH fluctuations are followed swiftly by corresponding changes in −ΔG. Circles on the y axis show the −ΔG values at stasis at pHacidic 7. Histograms in (C) show the frequency distributions for the corresponding curves in (B), with the vertical lines denoting the values for stasis at pH 7 (solid black) and mean of the corresponding curve (dashed grey). (D) Although responses are somewhat slower, addition of 5% SPAP makes fluctuations more survivable by increasing power overall. (E) is analogous to (C). See S4 Fig. for similar fluctuations in the alkaline side.
(TIF)

S4 Fig Effect of fluctuations in external alkaline pH, while holding external acidic pH constant at pH 7. Qualitatively similar behavior to that of S3 Fig. was observed when fluctuations occur on the alkaline side.
(TIF)

S5 Fig Pumping in the presence of SPAP facilitates adaptation to more acidic regions. All three curves show a steady increase in −ΔG with 5% pump in equivalent pH gradients (each of 3 pH units) with decreasing membrane permeability. In relatively alkaline conditions (pH 7:10 and 6:9) the benefit of pumping increases with decreasing permeability, but is relatively modest. In more acidic environments (pH 5:8) there is initially a relatively greater payback to pumping as membrane permeability decreases. The reason is that at high membrane permeability (10^−2^ cm/s) and relatively acidic pH (5:8), there is a fast influx of H^+^ (from the acidic side) and a slow influx of OH^−^ (from the alkaline side), leading to the collapse of −ΔG. Pumping across a very leaky membrane gives little benefit even with SPAP (−ΔG is very low). Lowering membrane permeability limits H^+^ influx and enhances the benefits of pumping, giving a greater relative benefit in acidic conditions (pH 5:8). In contrast, with tight membranes (10^−6^ cm/s), cells are powered almost exclusively by their own pumps, with little contribution from the external gradient (−ΔG collapses in the absence of a pump; see Fig. 3A and 3B). Cells in relatively alkaline (6:9 and 7:10) environments now gain slightly more from pumping. The reason is that the opposing external H^+^ concentration is greater at pH 5:8, so pumping H^+^ out is harder than at pH 6:9 or 7:10. The figure thus shows a transition from a highly permeable gradient-powered system on the left to a low permeability pump-powered system on the right.
(TIF)

S1 Manuscript Corrected article. (DOCX)
