# Supplementary material for: A Bioenergetic Basis for Membrane Divergence in Archaea and Bacteria
Source: PLoS Biol. 2014 Aug 12;12(8):e1001926. doi: 10.1371/journal.pbio.1001926 (PMC4130499; doi:10.1371/journal.pbio.1001926)
Supplement: Table S1 — Parameters in the model and references. (DOC) [file pbio.1001926.s006.doc]

Table S1. Parameters in the model and references

| **Parameter** | **Value** | **Comment** | **Ref.** |
| --- | --- | --- | --- |
| **Concentrations^*^** | **[M]** |  |  |
| H^+^_ocean_ | 10^-7^ | pH 7. May have been as low as pH 5 | [66] |
| H^+^_vent_ | 10^-10^ | pH 10. Can be as high as pH 11 at present | [66] |
| Na^+^ | 0.4 | Could have been as high as 0.8 M | [67] |
| K^+^ | 0.01 | Could have been as high as 0.02 M | [67] |
| Cl^–^ | 0.41 | Chosen to balance out the concentrations of Na^+^ and K^+^ | [67] |
| H_2_ | 0.015 | Can be as high as 0.02 M | [68] |
| **Permeabilities^†^** | **[cm/s]** |  |  |
| H^+^ | 10^–3^ | Default value unless otherwise noted | [31] |
| OH^–^ | 10^–3^ | Assumed equal to H^+^ | [31] |
| Na^+^ | 10^–9^ | In general, six orders of magnitude less permeable than H^+^ | [31] |
| K^+^ | 10^–9^ | Assumed equally permeable to Na^+^ | [31,69] |
| Cl^–^ | 10^–7^ | In general, two orders of magnitude more permeable than Na^+^ | [70] |
| **Turnover rates** | **[s^-1^]** |  |  |
| ATPase | 270 | Parameterized from mitochondria | [71,72] |
| Ech | 700 | Parameterized from a soluble NiFe hydrogenase | [73] |
| SPAP | 1500 | Parameterized from *E. coli*’s NhaA SPAP | [74] |
| Pump | 200 | Parameterized from mitochondrial Complex I | [75] |
| **Surface areas** | **[m^2^]** |  |  |
| ATPase F_o_ subunit | 4·10^-17^ | Estimated from RCSB PDB entry: 1C17 | [20,72] |
| Ech | 3·10^-17^ | Relevant subunits of Complex I, estimated from PDB:4HEA | [76,77] |
| SPAP | 1.5·10^-17^ | Estimated from PDB:1ZCD | [74] |
| Pump | 3·10^-17^ | Assumed to be similar to Ech |  |
| **Others** |  |  |  |
| H^+^ per ATP | 3.33 | This many H^+^ enter the ATPase in the synthesis of 1 ATP | [27,78] |
| Protocell diameter | 1μm | Small diameter of *E. coli* | [79] |
| Temperature | 298.15 K | Standard temperature |  |
| Embedment | 50% | Protocell is exactly half-embedded in the alkaline side |  |

**^*^** Excluding H^+^ and OH^–^, all concentrations were assumed equal in the alkaline and acidic sides.

**^†^** In all simulations Na^+^ and Cl^–^ permeabilities were kept respectively six and four orders of magnitude higher than the permeability of H^+^.

20. Stock D, Leslie A, Walker J (1999) Molecular architecture of the rotary motor in ATP synthase. Science 286: 1700–1705.

27. Nicholls DG, Ferguson SJ (2013) Bioenergetics. Fourth Ed. London: Academic Press.

31. Deamer D, Bramhall J (1986) Permeability of lipid bilayers to water and ionic solutes. Chem Phys Lipids 40: 167–188.

66. Arndt NT, Nisbet EG (2012) Processes on the young Earth and the habitats of early life. Annu Rev Earth Planet Sci 40: 521–549.

67. Pinti D (2005) The origin and evolution of the oceans. Lect Astrobiol I: 83–112.

68. Proskurowski G, Lilley MD, Kelley DS, Olson EJ (2006) Low temperature volatile production at the Lost City Hydrothermal Field, evidence from a hydrogen stable isotope geothermometer. Chem Geol 229: 331–343.

69. Deamer D, Dworkin J (2005) Chemistry and physics of primitive membranes. Top Curr Chem 259: 1–27.

70. Nichols J, Deamer D (1980) Net proton-hydroxyl permeability of large unilamellar liposomes measured by an acid-base titration technique. Proc Natl Acad Sci USA 77: 2038–2042.

71. Etzold C, Deckers-Hebestreit G, Altendorf K (1997) Turnover number of *Escherichia coli* F0F1 ATP synthase for ATP synthesis in membrane vesicles. Eur J Biochem 243: 336–343.

72. Yoshida M, Muneyuki E, Hisabori T (2001) ATP synthase-a marvellous rotary engine of the cell. Nat Rev Mol Cell Biol 2: 669–677.

73. Liebgott P-P, Leroux F, Burlat B, Dementin S, Baffert C, et al. (2010) Relating diffusion along the substrate tunnel and oxygen sensitivity in hydrogenase. Nat Chem Biol 6: 63–70.

74. Hunte C, Screpanti E, Venturi M, Rimon A, Padan E, et al. (2005) Structure of a Na+/H+ antiporter and insights into mechanism of action and regulation by pH. Nature 435: 1197–1202.

75. Vinogradov AD (1998) Catalytic properties of the mitochondrial NADH-ubiquinone oxidoreductase (complex I) and the pseudo-reversible active/inactive enzyme transition. Biochim Biophys Acta 1364: 169–185.

76. Baradaran R, Berrisford JM, Minhas GS, Sazanov LA (2013) Crystal structure of the entire respiratory complex I. Nature 494: 443–448.

77. Marreiros BC, Batista AP, Duarte AMS, Pereira MM (2013) A missing link between complex I and group 4 membrane-bound [NiFe] hydrogenases. Biochim Biophys Acta 1827: 198–209.

78. Ferguson SJ (2010) ATP synthase: from sequence to ring size to the P/O ratio. Proc Natl Acad Sci USA 107: 16755–16756.

79. Moran U, Phillips R, Milo R (2010) SnapShot: key numbers in biology. Cell 141: 1262–1262.e1.
