# Supplementary material for: A Bioenergetic Basis for Membrane Divergence in Archaea and Bacteria
Source: PLoS Biol. 2014 Aug 12;12(8):e1001926. doi: 10.1371/journal.pbio.1001926 (PMC4130499; doi:10.1371/journal.pbio.1001926)
Supplement: Table S2 — BLAST-search results for matches of the archaeal M. jannaschii Mj1275 SPAP to at least one member of each of the 37 known prokaryotic phyla. (DOC) [file pbio.1001926.s007.doc]

**Table S2. BLAST-search results for matches of the archaeal *M. jannaschii* Mj1275 SPAP to at least one member of each of the 35 known prokaryotic phyla**

| **Phylum** | **G.I.** | **Description** | **%id** | **S** | **E** |
| --- | --- | --- | --- | --- | --- |
| **Archaea** |  |  |  |  |  |
| Euryarchaeota* | 294496655 | sodium/proton-potassium antiporter | 30.57 | 157 | 3.00e-42 |
| Thaumarchaeota | 563488844 | putative Na(+)/H(+) antiporter | 28.72 | 146 | 2.00e-39 |
| Nanohaloarchaeota | 339757263 | Kef-type K+ transport family | 25.2 | 96.3 | 3.00e-23 |
| Korarchaeota | 170290145 | sodium/hydrogen exchanger | 25.44 | 91.7 | 1.00e-21 |
| Crenarchaeota | 352683176 | Na(+)/H(+) antiporter | 27.85 | 95.9 | 2.00e-21 |
| Parvarchaeota | 255513731 | sodium/hydrogen exchanger | 24.27 | 83.6 | 9.00e-19 |
| Nanoarchaeota**^†^** | 516994317 | hypothetical protein: Na(+)/H(+) exchanger family | 28.95 | 52.0 | 2.00e-08 |
| **Bacteria** |  |  |  |  |  |
| Cyanobacteria | 515885330 | hypothetical protein. Sodium/hydrogen exchanger family | 30.59 | 168 | 7.00e-45 |
| Firmicutes | 15893735 | Na/H antiporter NapA | 33.51 | 160 | 2.00e-42 |
| Bacteroidetes-Chlorobi | 548235349 | putative uncharacterized protein . Sodium/hydrogen exchanger family | 30.34 | 149 | 1.00e-37 |
| β-Proteobacteria | 490375968 | Na+/H+ antiporter | 28.12 | 140 | 2.00e-37 |
| δ-Proteobacteria | 493978264 | Kef-type K+ transport system, membrane component. Sodium/hydrogen exchanger family | 27.89 | 135 | 3.00e-34 |
| Deinococcus-Thermus | 297624885 | sodium/hydrogen exchanger | 28 | 130 | 2.00e-32 |
| Chloroflexi | 156742237 | sodium/hydrogen exchanger | 26.67 | 122 | 9.00e-31 |
| Spirochaetes | 517350815 | hypothetical protein. Sodium/hydrogen exchanger family | 27.13 | 125 | 1.00e-29 |
| ε-Proteobacteria | 390940331 | Kef-type K+ transport system membrane protein. Sodium/hydrogen exchanger family | 28.75 | 122 | 1.00e-29 |
| Aquificae | 225849059 | Na+:H+ antiporter, NhaA family | 27.66 | 115 | 6.00e-29 |
| Elusimicrobia | 189485528 | NapA type Na+/H+ antiporter | 27.55 | 111 | 1.00e-28 |
| Fibrobacteres-Acidobacteria | 522212591 | hypothetical protein. Sodium/hydrogen exchanger family | 25.68 | 114 | 5.00e-28 |
| γ-Proteobacteria | 495083969 | sodium/hydrogen exchanger | 27.79 | 120 | 6.00e-28 |
| Fusobacteria | 310779803 | sodium/hydrogen exchanger | 27.35 | 116 | 2.00e-27 |
| Nitrospirae | 206891081 | Na/H+ antiporter | 28.23 | 110 | 3.00e-27 |
| Thermodesulfobacteria | 551229848 | sodium:proton antiporter | 26.72 | 106 | 3.00e-26 |
| Chlamydiae-Verrucomicrobia | 494656847 | sodium/hydrogen exchanger | 26.08 | 109 | 4.00e-26 |
| Synergistetes | 357419296 | transporter, CPA2 family. Sodium/hydrogen exchanger family | 27.34 | 108 | 2.00e-25 |
| Actinobacteria | 501185687 | putative Na+/H+ antiporter CPA2 family | 26.67 | 112 | 5.00e-25 |
| Planctomycetes | 283779895 | sodium/hydrogen exchanger | 25.18 | 102 | 2.00e-23 |
| Chrysiogenetes | 317050984 | sodium/hydrogen exchanger | 26.08 | 94.7 | 1.00e-22 |
| α-Proteobacteria | 334343506 | sodium/hydrogen exchanger | 27.88 | 100 | 8.00e-22 |
| Deferribacteres | 555548272 | hypothetical protein. Sodium/hydrogen exchanger family | 28.2 | 90.5 | 2.00e-20 |
| Gemmatimonadetes | 226226447 | putative sodium/hydrogen transporter | 25.92 | 68.9 | 2.00e-13 |
| Nitrospinae | 491148743 | Kef-type potassium transporter | 25.07 | 60.5 | 2.00e-10 |
| Nitrospinae | 491148743 | Kef-type potassium transporter | 25.07 | 60.5 | 2.00e-10 |
| Thermotogae**^†^** | 389842970 | NhaP-type Na+(K+)/H+ antiporter | 24.8 | 35 | 0.061 |
| Tenericutes**^†^** | 493942442 | na(+)/h(+) antiporter | 24.81 | 36.2 | 0.073 |
| Caldiserica**^‡^** | 383788853 | putative formate dehydrogenase subunit alpha | 22.38 | 30.8 | 0.073 |
| Dictyoglomi**^†^** | 206901223 | glycyl-tRNA synthetase, beta subunit | 37.21 | 30 | 0.29 |

Only the highest-matching sequence for each clade is shown. Details of the meaning of each column below.

Phylum: each of the 37 known prokaryotic phyla, considering each of the proteobacteria separately. Three further archaeal clades have recently been reported but are not yet annotated in the NCBI-BLAST databases.

G.I.: unique NCBI/GenBank sequence identification number for the highest matching gene product.

Description: a brief summary of the annotated description for the highest matching protein found in the search.

%id: the percentage identity of the sequence to the Mj1275.

E: a measure of the likelihood of finding such a match with score S by chance.

S: the “bit score”. A measure of the quality of the alignment and match between the sequences.

**^*^** Euryarchaeota excluding the *Methanococcus* genus.

**^†^** These phyla produced a match to a SPAP, but the result is below the threshold of significance (E≤10^–10^).

**^‡^** These phyla produced unsuccessful results.

Only two bacterial clades (Caldiserica and Dictyoglomi) failed to give a match to a SPAP. During the development of this work, three previously negative matches were found, so it is possible that the two negative results are due to missing annotations or under-sampling. Additionally, the number of archaeal clades in the NCBI Taxonomy list ([ncbi.nlm.nih.gov/Taxonomy/Browser/wwwtax.cgi](http://www.ncbi.nlm.nih.gov/Taxonomy/Browser/wwwtax.cgi)) was duplicated from five to ten during the production of this work. The Mj1275 SPAP was positively matched to two of the five new clades (Nanohaloarchaeota and Parvarchaeota). The remaining three (Aenigmarchaeota, Diapherotrites, Geoarchaeota) have not been annotated into NCBI-BLAST at the time of writing. As described in the main text, the two bacterial clades that gave a negative result have only one representative member species, which may well have lost the gene or not yet have been properly annotated. Two further bacterial clades (Thermotogae and Tenericutes) do contain an Mj1275-matching SPAP gene, but the result has an E value larger than 10^–10^, considered an acceptable cut-off for deep phylogenetics [13], as is the case for the archaeal Nanoarchaeota. All of these three phyla also have a single member species.
