# Supplementary figures and images for: Correction: A Bioenergetic Basis for Membrane Divergence in Archaea and Bacteria
Source: PLoS Biol. 2015 Mar 20;13(3):e1002102. doi: 10.1371/journal.pbio.1002102 (PMC4368600; doi:10.1371/journal.pbio.1002102)

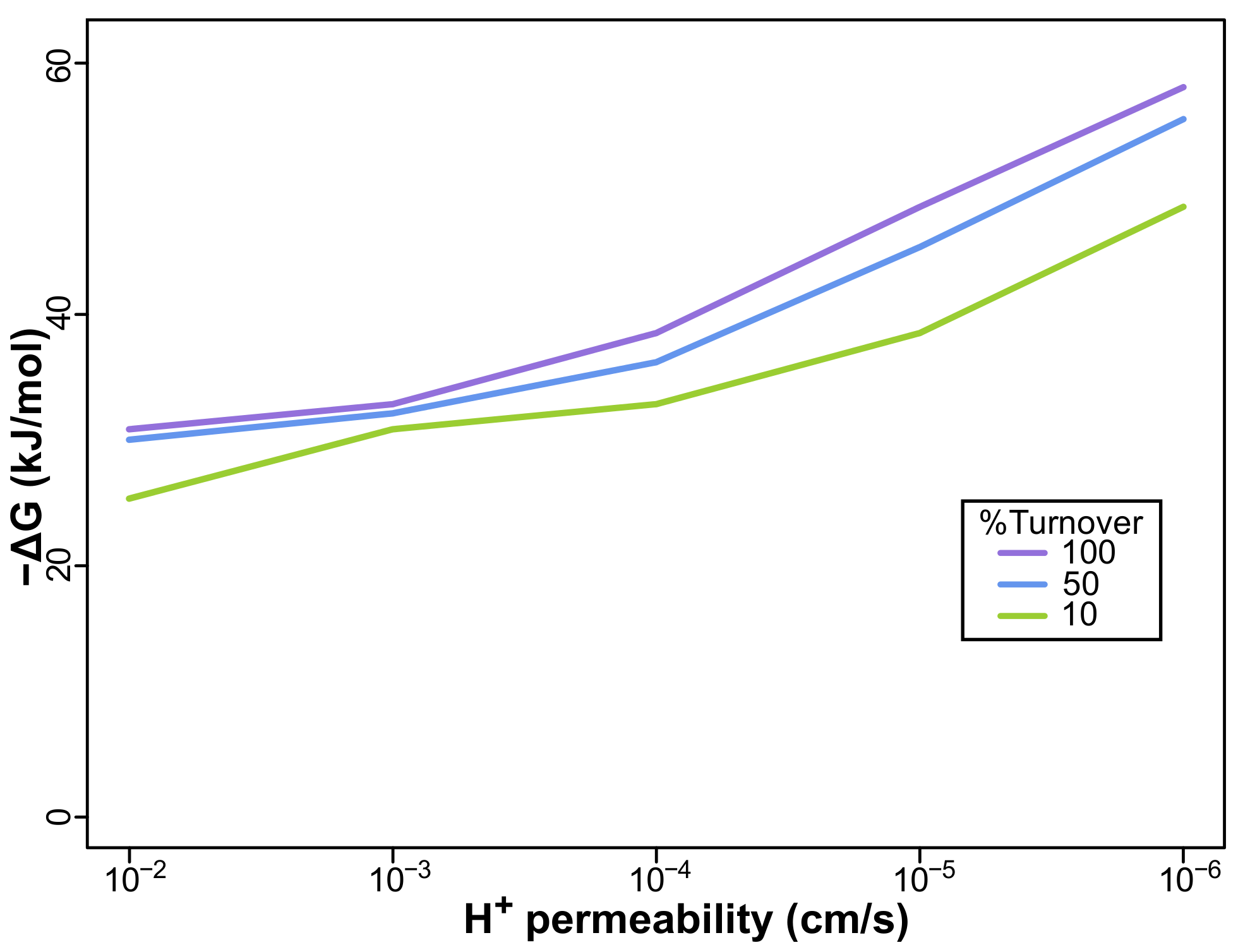

Supplement: S1 Fig — We assume that membrane proteins in LUCA had lower turnover rates than those in modern archaea and bacteria. For all the results in the main text, turnover rates were modeled at 10% of modern values (see S1 Table for these values). The figure shows that with ATPase, SPAP, and pump, the behavior is similar when turnover is set at 10%, 50%, and 100% for each protein. Parameters: 5% pump, 1% ATPase, 1% SPAP, pH gradient 7:10. (TIF) [file pbio.1002102.s001.tif]

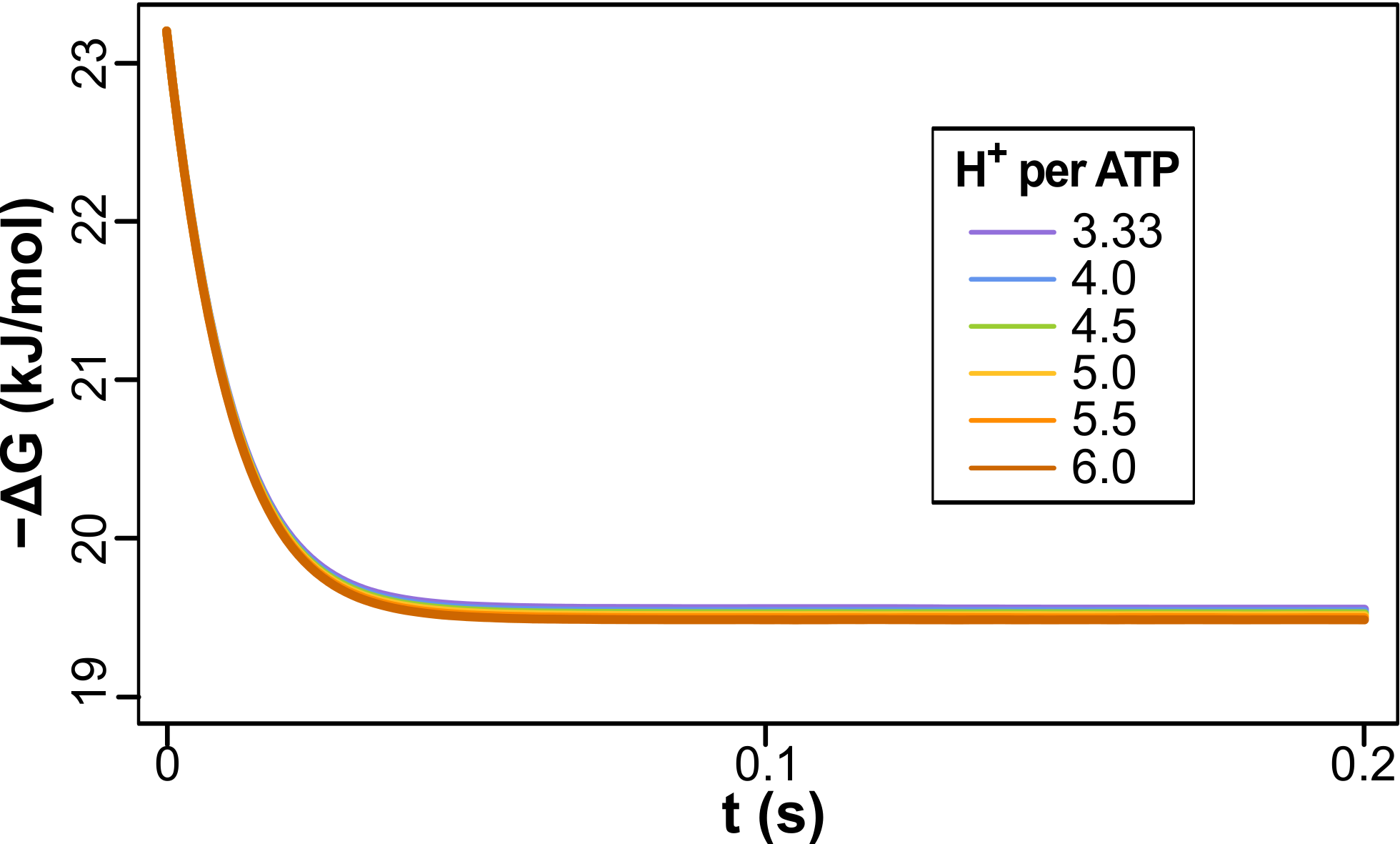

Supplement: S2 Fig — Lowering the efficiency of the ATPase by increasing the number of H+ necessary to synthesize one ATP molecule has a minor effect on the simulation results. Almost halving efficiency to 6 H+ per ATP lowers −ΔG by less than 1%. (TIF) [file pbio.1002102.s002.tif]

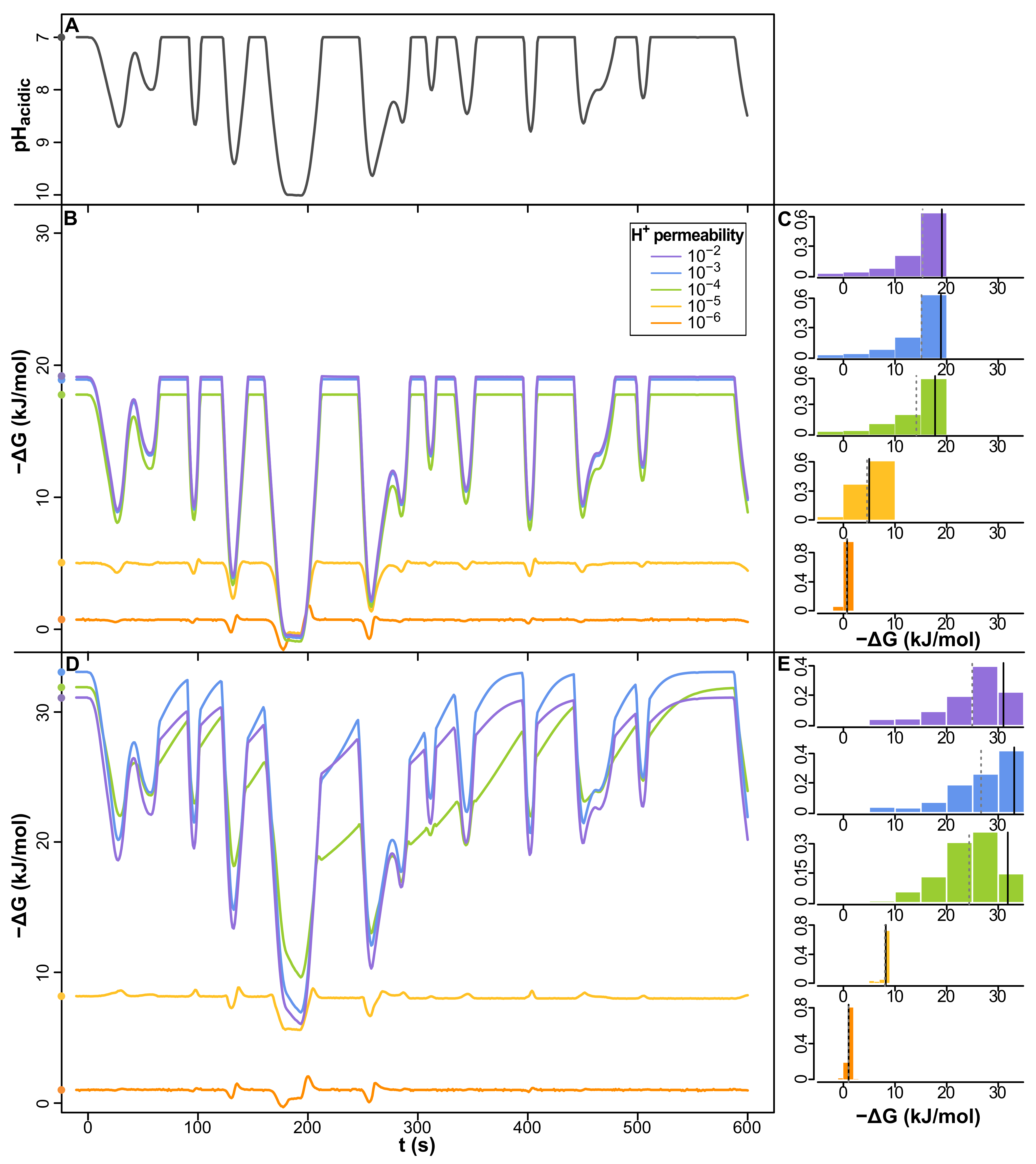

Supplement: S3 Fig — We considered the effect of mixing, with alkaline fluids causing local fluctuations in the pH of the acidic side. These were taken to occur on a scale of seconds, causing meaningful perturbations to the pH gradient and −ΔG. (A) Increases in the pH of the acidic side shrink the exploitable gradient. (B) With 1% ATPase and no SPAP or pump in the membrane, pH fluctuations are followed swiftly by corresponding changes in −ΔG. Circles on the y axis show the −ΔG values at stasis at pHacidic 7. Histograms in (C) show the frequency distributions for the corresponding curves in (B), with the vertical lines denoting the values for stasis at pH 7 (solid black) and mean of the corresponding curve (dashed grey). (D) Although responses are somewhat slower, addition of 5% SPAP makes fluctuations more survivable by increasing power overall. (E) is analogous to (C). See S4 Fig. for similar fluctuations in the alkaline side. (TIF) [file pbio.1002102.s003.tif]

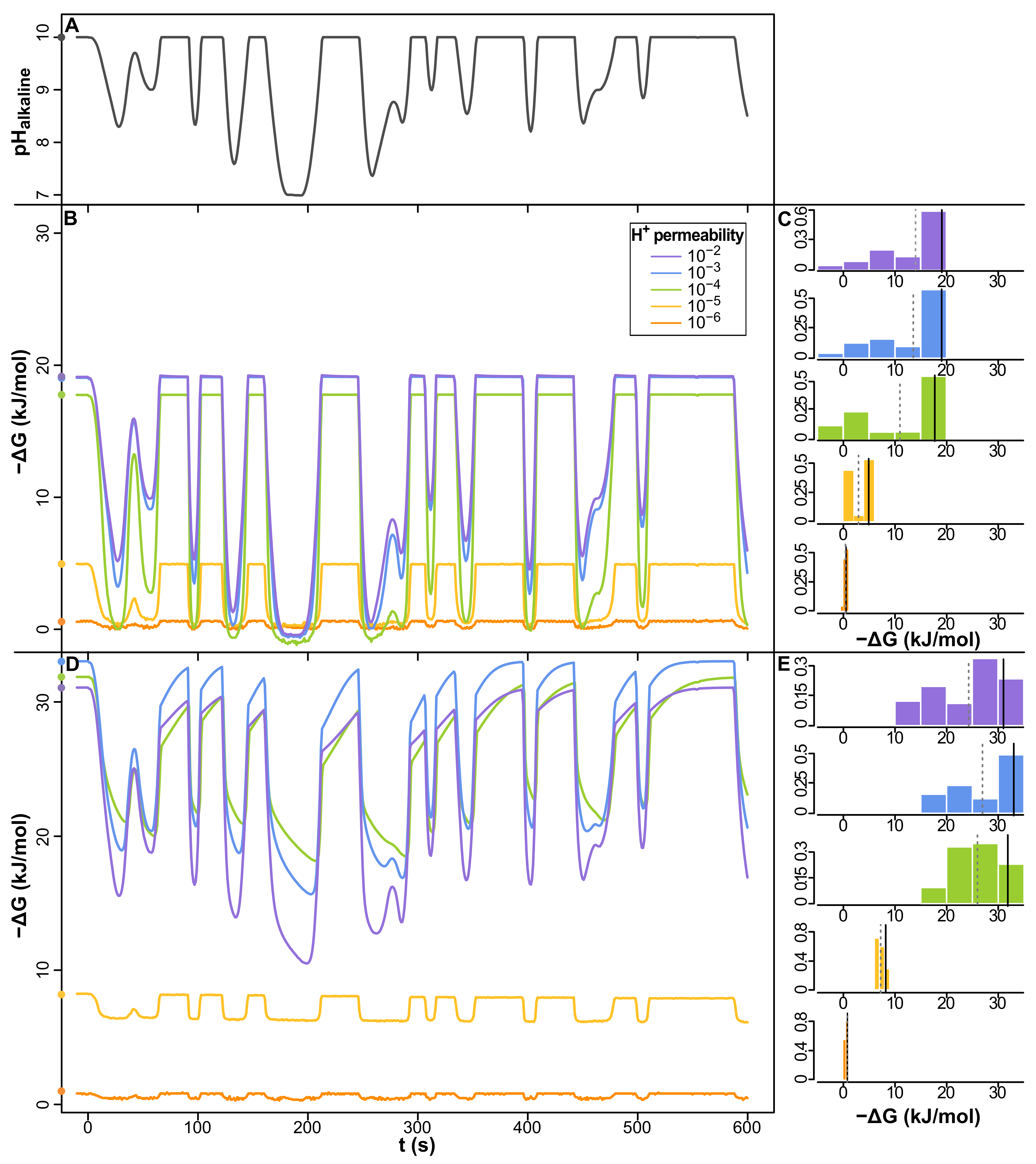

Supplement: S4 Fig — Qualitatively similar behavior to that of S3 Fig. was observed when fluctuations occur on the alkaline side. (TIF) [file pbio.1002102.s004.tif]

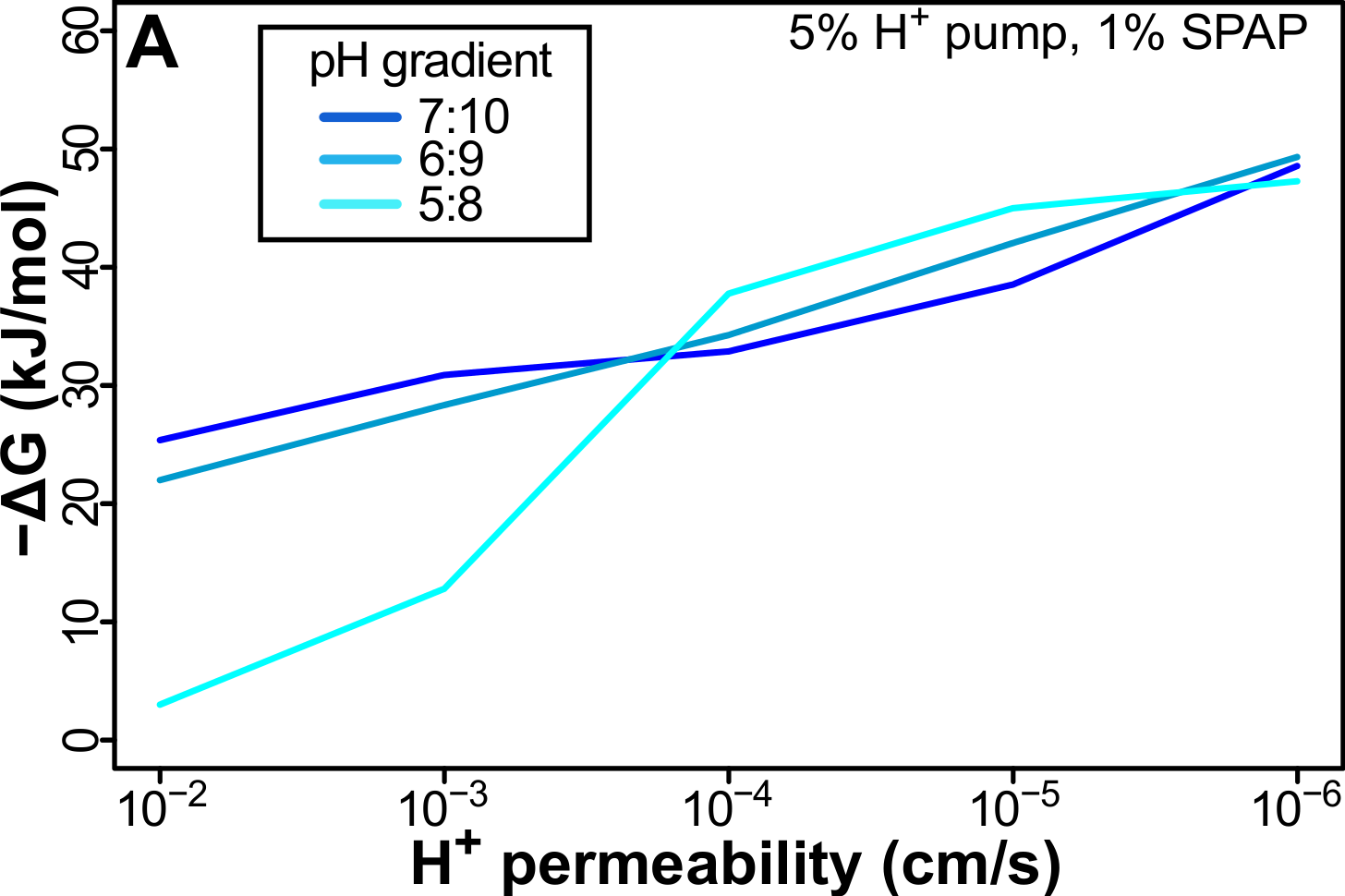

Supplement: S5 Fig — All three curves show a steady increase in −ΔG with 5% pump in equivalent pH gradients (each of 3 pH units) with decreasing membrane permeability. In relatively alkaline conditions (pH 7:10 and 6:9) the benefit of pumping increases with decreasing permeability, but is relatively modest. In more acidic environments (pH 5:8) there is initially a relatively greater payback to pumping as membrane permeability decreases. The reason is that at high membrane permeability (10−2 cm/s) and relatively acidic pH (5:8), there is a fast influx of H+ (from the acidic side) and a slow influx of OH− (from the alkaline side), leading to the collapse of −ΔG. Pumping across a very leaky membrane gives little benefit even with SPAP (−ΔG is very low). Lowering membrane permeability limits H+ influx and enhances the benefits of pumping, giving a greater relative benefit in acidic conditions (pH 5:8). In contrast, with tight membranes (10−6 cm/s), cells are powered almost exclusively by their own pumps, with little contribution from the external gradient (−ΔG collapses in the absence of a pump; see Fig. 3A and 3B). Cells in relatively alkaline (6:9 and 7:10) environments now gain slightly more from pumping. The reason is that the opposing external H+ concentration is greater at pH 5:8, so pumping H+ out is harder than at pH 6:9 or 7:10. The figure thus shows a transition from a highly permeable gradient-powered system on the left to a low permeability pump-powered system on the right. (TIF) [file pbio.1002102.s005.tif]
